# Tunable Chemical and Optical Modulation of ER-Plasma Membrane Contact-Site Geometry, Dynamics, and Protein Recruitment with High-Fidelity Visualization

**DOI:** 10.1021/acs.analchem.6c03857

**Published:** 2026-08-11

**Authors:** Shaoqing Zhang, Jinyu Fei, Yuanmin Zheng, Ruobo Zhou

**Affiliations:** † Department of Chemistry, 8082 The Pennsylvania State University, University Park, Pennsylvania 16802, United States; ‡ Department of Biomedical Engineering, The Pennsylvania State University, University Park, Pennsylvania 16802, United States; § Department of Biochemistry and Molecular Biology, The Pennsylvania State University, University Park, Pennsylvania 16802, United States; ∥ The Huck Institutes of Life Sciences, The Pennsylvania State University, University Park, Pennsylvania 16802, United States

## Abstract

Endoplasmic reticulum–plasma membrane (ER–PM) contact sites are dynamic membrane interfaces that regulate essential cellular processes, including calcium signaling and lipid homeostasis. Emerging evidence suggests that contact-site geometry and organization critically influence these functions, yet existing tools cannot systematically manipulate individual geometric parameters while providing high-fidelity visualization in living cells. Here, we develop complementary chemical and optical platforms for inducible ER–PM contact-site reconstitution. A nontoxic, reversible abscisic acid-inducible system based on the plant-derived ABIcs/PYLcs pair and a rapidly reversible optogenetic iLID/SspB system enable robust visualization and dose-dependent control of contact-site assembly and disassembly. Increasing inducer dose selectively increases contact-site density and total contact area per cell without substantially changing the average size of individual contact sites. In contrast, systematic variation of tether length or tether abundance selectively increases average contact-site size and total contact area without altering contact-site density, providing orthogonal strategies to modulate contact-site density and size. Importantly, engineered contact sites recruit ER–PM contact-site-associated proteins, including MAPPER, Kv2.1, and Stromal Interaction Molecule 1 (STIM1), demonstrating key organizational features of native ER–PM contacts. MAPPER recruitment is maintained across tether lengths tested, whereas Kv2.1/STIM1 recruitment depends strongly on tether length, with longer tethers promoting recruitment by increasing intermembrane spacing. In contrast, varying tether abundance has little effect on protein recruitment, revealing distinct roles for intermembrane spacing and tether density in organizing ER–PM contact sites. Together, this work establishes a quantitative platform for engineering ER–PM contact sites with defined geometric properties and provides a framework for investigating how membrane contact-site architecture regulates cellular function.

## Graphical abstract

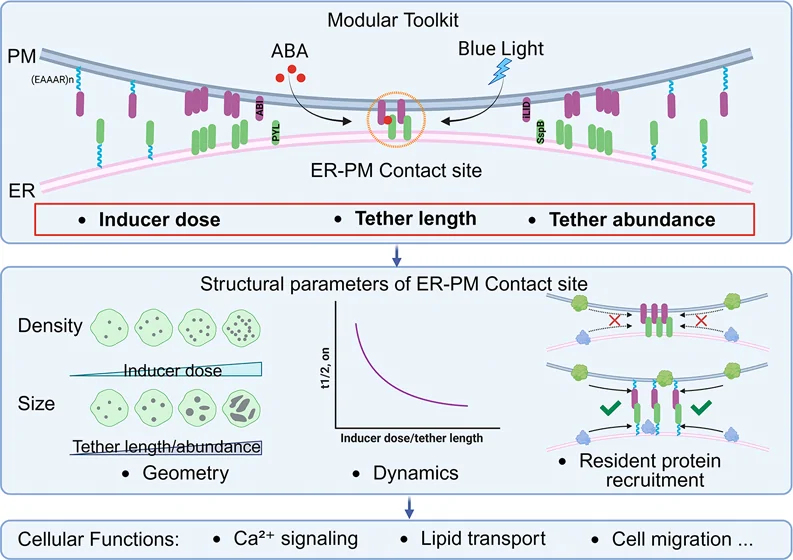

## Introduction

Membrane contact sites (MCSs) are regions where two organelles are closely apposed without membrane fusion, maintained by dedicated protein- and lipid-based tethers in eukaryotic cells.^1^ MCSs support a broad array of essential cellular functions, including non-vesicular lipid transport, Ca^2+^ and ion homeostasis, organelle positioning and dynamics, metabolic coordination, signaling compartmentalization, autophagy regulation, and cellular stress responses.^2^ Both mammalian cells and budding yeast are well-established model systems for MCS research.^1^ Among the diverse classes of MCSs, endoplasmic reticulum–plasma membrane (ER–PM) contact sites constitute one of the most abundant and functionally versatile MCS types in eukaryotic cells,^3^ which is a highly regulated subcellular interface that supports lipid transfer, Ca^2+^ signaling and homeostasis, phosphoinositide metabolism, cell signaling polarity, cell migration, and membrane repair.^4^ Dysregulation of ER–PM contact sites has been implicated in numerous pathological conditions, including neurodegenerative disorders,^5^ muscular hypotonia and related myopathies,^6^ immune deficiencies,^7^ and cardiovascular and metabolic abnormalities.^8^ A well-characterized example of ER–PM function is Ca^2+^ signaling: depletion of ER Ca^2+^ activates the ER-resident Ca^2+^ sensor Stromal Interaction Molecule 1 (STIM1), which translocates to ER–PM junctions and engages the PM-resident Orai1 Ca^2+^ channel to drive store-operated calcium entry (SOCE).^9^ ER–PM contacts thereby regulate Orai1-dependent SOCE to support muscle growth and reduce fatigue in adult skeletal muscle.^10^ In addition, beyond their mere formation, ER–PM contact sites are increasingly recognized as tunable physical interfaces whose nanoscale geometry and dynamics may influence functional specificity.^11–14^ Native ER–PM contacts span a broad range of intermembrane distances, sizes, densities, and lifetimes, and these geometric parameters vary across cell types, subcellular regions, and physiological states. Accumulating evidence suggests that such geometric heterogeneity is not incidental but functionally consequential, influencing calcium signaling,^12^ cytoskeletal coupling,^13^ and membrane mechanics.^14^ In migrating cells, ER–PM contacts display front-rear asymmetry, with small transient contacts at the leading edge and larger stable contacts at the trailing edge, contributing to directional signaling and migration.^11^ Collectively, given their indispensable roles in lipid exchange, Ca^2+^ dynamics, and cellular signaling, precise tools for visualizing and acutely manipulating ER–PM contact sites in living cells are needed to investigate how their organization and geometry regulate cellular physiology.

Although several molecular tools have been developed for either visualizing or manipulating ER–PM contact sites, existing systems typically provide either reversible control or contact-site-specific visualization, but not both simultaneously:^15–17^ (1) Genetically encoded ER–PM contact-site marker MAPPER^15^ (“membrane-attached peripheral ER”) fuses an ER-targeting sequence with a linker and a phosphoinositide-binding motif from the Rit polybasic (PB) domain to associate with the PM. While useful for labeling ER–PM contacts, MAPPER cannot manipulate the dynamic formation and dissociation of ER–PM contacts, and its constitutive tethering could potentially perturb endogenous phosphoinositide homeostasis.^18^ (2) Optogenetic approaches, such as LiMETER, are based on the MAPPER design and incorporate the AsLOV2 light-sensing domain upstream of the Rit-PB domain.^16^ In the dark, AsLOV2 inhibits Rit-PB binding to the plasma membrane, whereas blue light illumination triggers Rit-PB–PM interaction, enabling reversible, noninvasive, and precise manipulation of ER–PM contacts. However, because the LiMETER construct is fluorescently tagged and anchored to the dense reticular ER network and remains broadly distributed across the ER after induction, the induced ER–PM contact sites are partially obscured by the surrounding ER signal, making them difficult to visually distinguish from the ER mesh. (3) Chemically inducible dimerization (CID) systems provide an alternative strategy to control ER–PM contact-site formation with small-molecule inducers. The rapamycin-based FRB-FKBP system has been used to tether the ER and PM and to demonstrate that ER–PM contacts serve as platforms for STIM1–Orai1 interactions during store-operated Ca^2+^ entry.^17^ Despite its utility in inducing heterodimerization between FKBP-tagged and FRB-tagged proteins, rapamycin-based CID is limited by extremely slow dissociation kinetics,^19^ interference with endogenous mTOR signaling,^20^ high cost, potential cytotoxicity,^21^ and reduced specificity due to competition with endogenous FKBP or cyclophilin family proteins.^22^ Together, these limitations highlight the need for tools that enable both reversible manipulation and high-fidelity visualization of ER–PM contact sites for quantitative analysis of their dynamic functions.

Here, we present a modular toolkit enabling simultaneous high-fidelity visualization and reversible, tunable control of ER–PM contact sites in living cells, comprising two complementary inducible tethering platforms: a chemically inducible system based on the plant-derived abscisic acid (ABA)-dependent ABIcs/PYLcs interaction,^23^ and an optogenetic system based on the blue-light-responsive iLID/SspB pair.^24^ The ABA-based system offers nontoxic and reversible chemical control without interference from endogenous mTOR signaling, whereas the iLID/SspB system provides fast, noninvasive, reversible optical control with high temporal precision. Beyond inducible formation, we introduce orthogonal, design-driven strategies to modulate contact-site architecture: tuning ABA concentration or blue-light intensity primarily controls contact-site density and total cellular contact area without altering the mean size of contacts, whereas systematic variation of rigid α-helical linker length (inserted as part of the tether) or inducible control of tether abundance selectively modulates the mean size of contacts without affecting their density. Importantly, using tunable linker constructs, we show that MAPPER, Kv2.1, and STIM1 display distinct recruitment behaviors across different intermembrane spacings: MAPPER is efficiently recruited across all spacings tested, whereas Kv2.1 and STIM1 exhibit spacing-dependent recruitment in which longer linkers (larger intermembrane spacing) progressively enhance recruitment, with STIM1 showing intermediate sensitivity and Kv2.1 being more strongly restricted at shorter distances, highlighting how contact-site geometry can differentially influence protein recruitment and potentially functional organization at ER–PM contacts. Together, these approaches provide independent and tunable modulation of the overall properties of ER–PM contact sites, including contact-site dynamics, geometry, extent, and recruitment of ER–PM contact site-resident proteins, offering a versatile framework for investigating how ER–PM contact-site organization and geometry contribute to cellular processes such as lipid transport and Ca^2+^ signaling.

## Experimental Section

### Plasmid Construction

Inducible ER–PM contact site formation systems were engineered using rapamycin (FRB/FKBP), abscisic acid (ABI/PYL), and blue light (iLID/SspB) dimerization pairs. PM and ER targeting was achieved using the N-terminal Lyn kinase signal and the C-terminal human Sac1 phosphatase sequence, respectively. The linker length was further tuned using (EAAAR)­n repeats. Detailed sequences and cloning strategies are provided in the Supporting Information (SI).

### Cell Culture and Induction Assays

U2OS and HEK293T cells were cultured and transfected according to standard protocols (see SI). Chemical induction was achieved using ABA- and rapamycin-based dimerization systems, while optogenetic control was implemented via blue-light illumination. Reversibility of ER–PM contact formation was evaluated by compound washout or light withdrawal, with dynamics monitored by live cell imaging.

### Microscopy and Image Analysis

Confocal and widefield epi-fluorescence imaging were performed as described in the SI. ER–PM contact site puncta were identified by using custom MATLAB scripts incorporating top-hat filtering, Otsu thresholding, and morphological refinement to generate binary masks for feature extraction. Contact site metrics, including area fraction, size, density, and fluorescence intensity, as well as protein colocalization, were quantified on the basis of mask-defined regions. Temporal kinetics were determined by fitting time-course data to single-exponential association and dissociation models to extract rate constants and half-times.

## Results and Discussion

### An ABA-Inducible CID System Enables Nontoxic, Tunable, and Reversible Control of ER–PM Contact-Site Formation

To overcome the limitations of currently available rapamycin-based ER–PM contact-forming CID systems, including irreversibility, cytotoxicity, interference with endogenous mTOR signaling, and reduced specificity due to competition with endogenous FKBP or cyclophilin family proteins in eukaryotic cells, we sought to develop a novel abscisic acid (ABA)-inducible CID system to chemically control ER–PM contact-site formation. CID systems based on ABA-induced heterodimerization between ABIcs- and PYLcs-tagged proteins represent a potentially advantageous alternative to rapamycin-based systems, as they are derived from plant signaling pathways and are therefore expected to exhibit reduced interference with host-cell signaling and improved molecular specificity.^23^ Guided by the design of a previously established rapamycin-inducible ER–PM contact-forming system based on FRB/FKBP interactions, we implemented an analogous ER–PM tethering strategy using the ABIcs/PYLcs pair to construct an ABA-inducible ER–PM contact-forming CID system. A rapamycin-inducible ER–PM tethering system was constructed in parallel as a reference. In both systems, identical PM- and ER-targeting sequences were used to localize the dimerization domains to opposing membranes such that ER–PM tethering differed only in the inducible dimerization module. For PM targeting, the N-terminal palmitoylation/myristoylation signal sequence of human Lyn kinase^25^ was fused to the N terminus of ABIcs or FRB tagged with mRFP. For ER targeting, the C-terminal ER localization sequence of human Sac1 phosphatase^17^ was fused to the C-terminus of PYLcs or FKBP tagged with mStayGold. In both systems, the PM- and ER-targeted fusion proteins were encoded within a single bicistronic expression cassette and separated by a self-cleaving T2A peptide, enabling coexpression of the two proteins from a single transcript while producing separate polypeptides through ribosomal skipping^26^ (Figure 1A).

**Figure 1:**
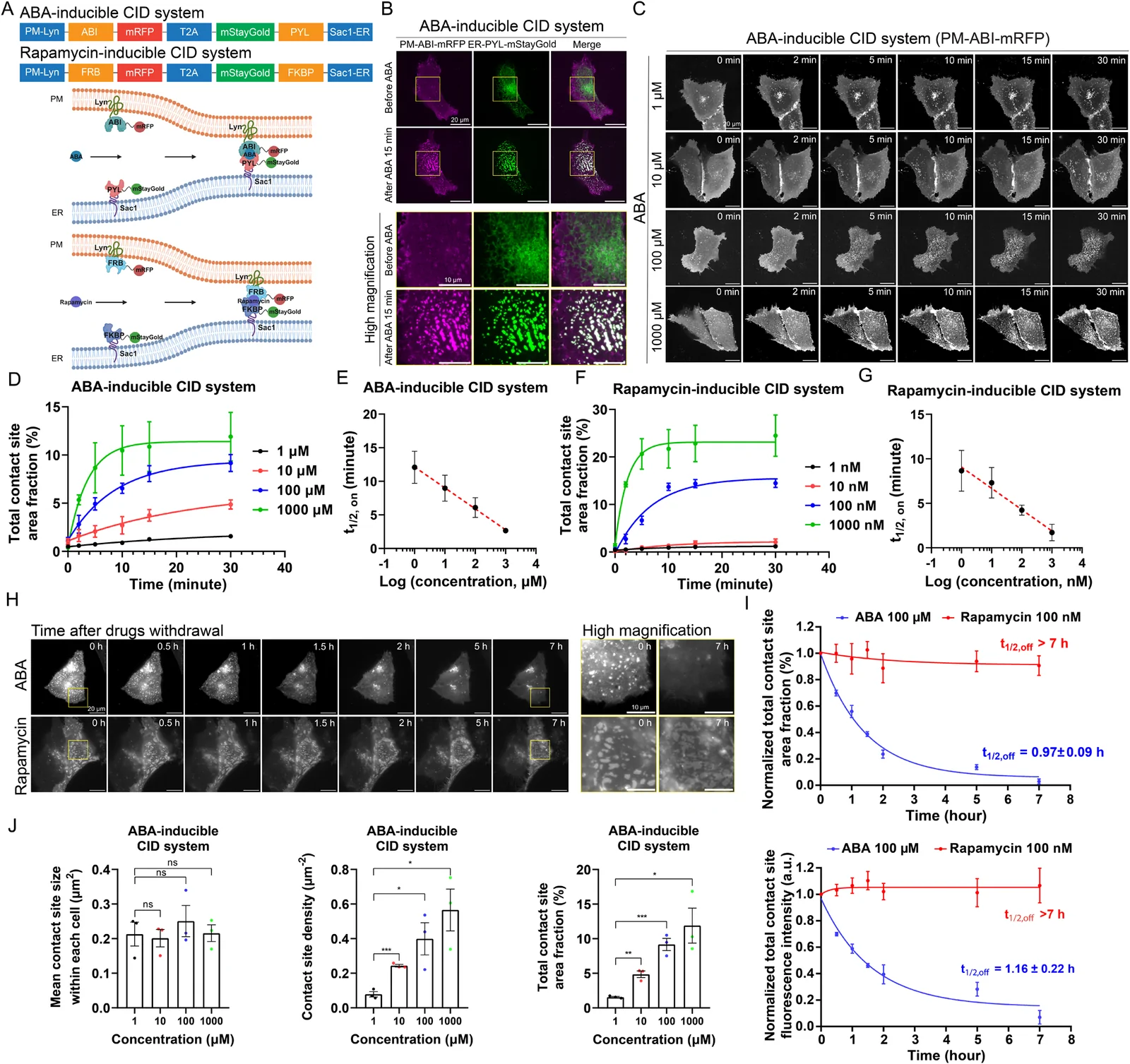
(A) Schematic of construct designs and ABA- and rapamycin-inducible CID systems. (B) Representative confocal images of U2OS cells expressing the ABA system before and after ABA treatment. (C) Time- and dose-dependent formation of ABA-induced ER–PM contact sites. (D, E) Quantification of the total contact-site area fraction and derived half-time (*t*_1/2,on_) fitted with a single-exponential association model. (F, G) Corresponding analysis for the rapamycin system. (H, I) Time course and quantification of contact site dissociation after drug withdrawal, fitted with a single-exponential dissociation model to calculate *t*_1/2,off_. (J) Quantification of contact site size, density, and area fraction at varying ABA concentrations. Data represent mean ± s.e.m. (*n* = 3). Scale bars: 20 μm (original view) and 10 μm (magnified view). *P* values were calculated using two-tailed unpaired Student’s *t*-test: *P < 0.05, **P < 0.01, ***P < 0.001, ****P < 0.0001, and ns, not significant.

We first validated whether the dimerization domains were correctly localized to their intended subcellular compartments and whether ABA could induce ER–PM contact-site formation in the designed ABA-inducible ER–PM contact-forming CID system (PM-ABIcs-mRFP-T2A-mStayGold-PYLcs-ER), using confocal microscopy. Prior to ABA addition, cells expressing this construct showed uniform plasma membrane localization of PM-ABIcs-mRFP, while mStayGold-PYLcs-ER displayed an ER network pattern, with minimal colocalization between the two fluorescence signals (Figure 1B). Upon ABA addition, PM-ABIcs-mRFP and mStayGold-PYLcs-ER rapidly reorganized into discrete punctate structures at the cell surface within 5 min, where they exhibited tight signal colocalization (Figure 1B). These puncta appeared as bright, well-defined foci with sharp boundaries, clearly distinct from the surrounding PM and ER regions. Cells expressing the rapamycin-inducible ER–PM contact-forming CID system (PM-FBR-mRFP-T2A-mStayGold-FKBP-ER) exhibited similar localization patterns before stimulation and comparable puncta formation following rapamycin treatment (Figure S1A), consistent with previous reports of rapamycin-induced ER–PM tethering.^17^ It is worth noting that, prior to induction, a small fraction of cells (∼15%) in both the ABA- and rapamycin-inducible systems exhibited intracellular punctate signal enrichment in addition to plasma membrane localization (Figure S1B). These pre-existing intracellular puncta did not colocalize with the genetically encoded ER–PM contact-site marker MAPPER, indicating that these structures do not represent pre-existing ER–PM contact sites prior to induction (Figure S1C). Instead, this occasional intracellular enrichment likely reflects partial endosomal localization of the Lyn-derived plasma membrane-targeting motif, as previously reported for Src-family kinase membrane anchors^27^ and can be excluded from the quantification. Together, these results demonstrate that the ABA-inducible CID system enables robust and specific induction and visualization of ER–PM contact sites, with behavior comparable to that of the established rapamycin-inducible system.^17^

Next, we compared the cytotoxicity of ABA and rapamycin treatments, as ABA is not expected to interfere with endogenous mTOR signaling pathways, unlike rapamycin. Cells were treated with increasing concentrations of ABA or rapamycin for 72 h, and cell viability was assessed using MTT assays. Rapamycin caused a dose-dependent decrease in viability (IC_50_ = 0.716 ± 0.358 μM), indicating substantial cytotoxicity, whereas ABA showed no detectable cytotoxic effects even at 1000 μM, with viability comparable to untreated controls (Figure S2A–C). The absence of cytotoxicity in the ABA system is consistent with its plant origin: ABA is a plant-derived phytohormone and ABA signaling is absent in mammalian cells, eliminating endogenous receptors or competing proteins. By contrast, rapamycin is a macrolide compound that potently inhibits the mTOR signaling pathway and exhibits well-documented cytostatic and cytotoxic effects across multiple mammalian cell types. Previous studies have shown that rapamycin induces growth inhibition or apoptosis in cell lines, such as HeLa, HEK293, U2OS, and MIN6, that are commonly used in ER–PM contact site studies.^21,28–30^ Furthermore, because the mTOR-rapamycin-FKBP12 pathway is endogenous to mammalian cells, competition from endogenous FKBP12 may reduce the efficiency of exogenous FKBP-fused constructs, potentially compromising dimerization specificity and experimental reproducibility.

To quantify the kinetics of ER–PM contact-site formation and assess whether it can be precisely controlled by ABA concentration, we monitored the time course of contact-site formation at multiple ABA doses (Figure 1C). During induction, the PM-anchored ABIcs-mRFP signal transitioned from a uniform plasma membrane distribution to discrete puncta, providing higher contrast than the mStayGold-PYLcs-ER signal, which reorganized from a reticular ER network and could lead to misidentification of ER structures as puncta. Therefore, PM-ABIcs-mRFP was used as the representative imaging channel for quantifying ER–PM contact sites. At each ABA concentration, the total area fraction of ER–PM contact sites, defined as the total contact-site area per cell normalized to total cell area, increased exponentially over time (Figure 1D). The induction half-time (*t*_1/2,on_) was extracted by fitting the data to a single exponential function. Kinetic analysis revealed a clear dose dependence of *t*_1/2,on_. Specifically, as ABA concentration increased in log10 increments (1, 10, 100, and 1000 μM), *t*_1/2,on_ decreased linearly with log10­[ABA] (Figure 1E), indicating an ABA concentration-dependent acceleration of ER–PM contact-site formation. A similar concentration-dependent kinetic behavior was observed for the rapamycin-induced CID system, in which *t*_1/2,on_ decreased linearly with increasing log10­[rapamycin] (1–1000 nM) (Figures 1F,G and S3A). Importantly, the predictable relationship between inducer concentration and induction kinetics enables precise and tunable control of ER–PM contact-site formation: using the predetermined calibration curves (Figure 1E,G), a defined chemical concentration can be applied to achieve a desired rate of contact-site formation.

This inducer (i.e., ABA, rapamycin) concentration-dependent acceleration may arise from mass-action kinetics,^31,32^ whereby increasing small-molecule inducer concentrations enhance the effective association rate of the CID pairs, or from receptor occupancy effects that yield a log–linear response to inducer concentration.^33^ Additionally, the trend may reflect modulation of a rate-limiting nucleation step during the ER–PM contact site assembly. The concurrent decrease in *t*_1/2,on_ and the increase in steady-state total contact-site area fraction at higher inducer concentrations suggest that ABA or rapamycin primarily regulates the frequency and probability of nucleation events rather than the lateral expansion rate of contact zones. At elevated inducer concentrations, the rapid formation of ABIcs/PYLcs or FRB/FKBP complexes likely increases the density of productive nucleation events at the ER–PM interface, leading to faster and more extensive contact site assembly. Together, these findings demonstrate that both the extent and kinetics of chemically induced ER–PM contact-site formation scale linearly with log_10_[inducer concentration]. Importantly, this tunable and predictable dose–response relationship enables systematic modulation of ER–PM contact dynamics, establishing a calibration-like framework analogous to analytical standard curves for dissecting chemically induced inter-organelle tethering dynamics.

To evaluate the reversibility of chemically induced ER–PM contact-site formation, we examined and compared the recovery kinetics of the ABA- and rapamycin-based CID systems following inducer withdrawal. Cells expressing either system were first treated with the corresponding inducer to fully establish ER–PM contact sites, after which the inducer was removed by washing the cells five times with a fresh inducer-free medium. Time-lapse imaging showed that ABA-induced ER–PM contact sites gradually disassembled and returned to baseline levels within ∼7 h after ABA removal (Figure 1H). In contrast, identical washout conditions failed to reverse rapamycin-induced ER–PM contacts over the same time period, indicating a marked difference in reversibility between the two systems. Quantification of the total ER–PM contact-site area fraction and total contact site fluorescence intensity per cell revealed recovery half-times (*t*_1/2,off_) of 0.97 ± 0.09 h for total contact-site area fraction and 1.16 ± 0.22 h for total contact site fluorescence intensity (Figure 1I) in the ABA system, whereas rapamycin-induced contacts showed *t*_1/2,off_ values exceeding 7 h. These differences likely reflect fundamental differences in molecular affinity and complex stability of the CID pairs. The ABIcs/ABA/PYLcs interaction exhibit lower affinity and faster dissociation kinetics^23^ than the high-affinity FRB/rapamycin/FKBP ternary complex,^19,34^ rendering ABA-induced ER–PM contacts readily reversible. This reversibility provides significant experimental advantages, including the ability to perform repeated induction–recovery cycles, dissect the kinetics of contact site nucleation versus expansion, and transiently perturb ER–PM contact-dependent processes without inducing long-lasting structural alterations.

Having established that the ABA-inducible system exhibits superior reversibility and nontoxicity compared to the rapamycin-inducible system, we next examined how steady-state ER–PM contact-site geometry responds to ABA concentration. Cells were treated with increasing ABA doses (1, 10, 100, and 1000 μM), and contact sites were allowed to reach the steady state after 30 min of induction. We then quantitatively analyzed three geometric parameters: the mean contact-site size (average area of individual contacts within each cell), contact-site density (number of contacts per unit cell area), and the total contact-site area fraction per cell. As ABA concentration increased logarithmically, both contact-site density and total contact-site area fraction increased monotonically, whereas the mean contact-site size remained largely unchanged across the tested concentration range (Figure 1J). Similar trends were also observed in the rapamycin-inducible system, in which increasing rapamycin concentrations increased contact-site density and total contact-site area fraction; however, unlike the ABA-inducible system, mean contact-site size also increased (Figure S3B). Construct fluorescence intensities showed no significant difference between the ABA- and rapamycin-inducible systems (Figure S3C), suggesting that the differences in contact-site size behavior between the two systems likely arise from differences in tether architecture rather than unequal construct expression. These results suggest that ABA concentration primarily controls ER–PM contact-site nucleation and abundance, whereas the lateral dimensions of contacts are constrained by the underlying tether architecture rather than inducer dose.

Together, our results demonstrate that the ABA-inducible CID system developed here, in contrast to rapamycin-based CID systems, enables nontoxic, tunable, and reversible manipulation and visualization of ER–PM contact sites with minimal interference from endogenous cellular pathways. These properties make the ABA system particularly well suited for dynamic live-cell imaging, quantitative biophysical analysis, and reversible control of inter-organelle interactions.

### An Optogenetic Light-Induced Dimerization (LID) System Enables Rapid, Tunable, and Reversible Control of ER–PM Contact-Site Formation

Though the ABA-inducible CID system developed here provides nontoxic, tunable, and reversible control of ER–PM contact-site formation and visualization, the recovery kinetics remain relatively slow with a recovery half-time (*t*_1/2,off_) of ∼1 h. To further accelerate reversibility, we turned to optogenetic tools, which enable genetically encoded, light-responsive modulation of protein interactions with high temporal precision in living cells. We therefore designed a dual-component LID system based on the iLID/SspB pair to induce ER–PM contact-site formation, where the iLID module is derived from the LOV2 domain of *Avena sativa* phototropin 1 and has been engineered for robust, rapid, and reversible blue-light-induced heterodimerization with SspB.^35,36^ Our design of the light-inducible ER–PM contact site forming LID system employed the same PM and ER targeting strategy as the ABA-inducible CID system: iLID was fused to a PM-targeting peptide (N-terminal Lyn motif) and tagged with mRFP, and SspB was fused to an ER-localization peptide (C-terminal Sac1 sequence) and tagged with mStayGold. The two fusion proteins were expressed using the same T2A-based bicistronic strategy as described for the CID system. In the dark, the Jα helix of iLID remains docked against the LOV2 core, sterically masking the embedded SsrA peptide and preventing its association with SspB. Upon blue-light illumination, LOV2 undergoes a conformational change that triggers undocking of the Jα helix, exposing SsrA and allowing its binding to ER-localized SspB, which in turn drives the assembly of ER–PM contact sites (Figure 2A).

**Figure 2:**
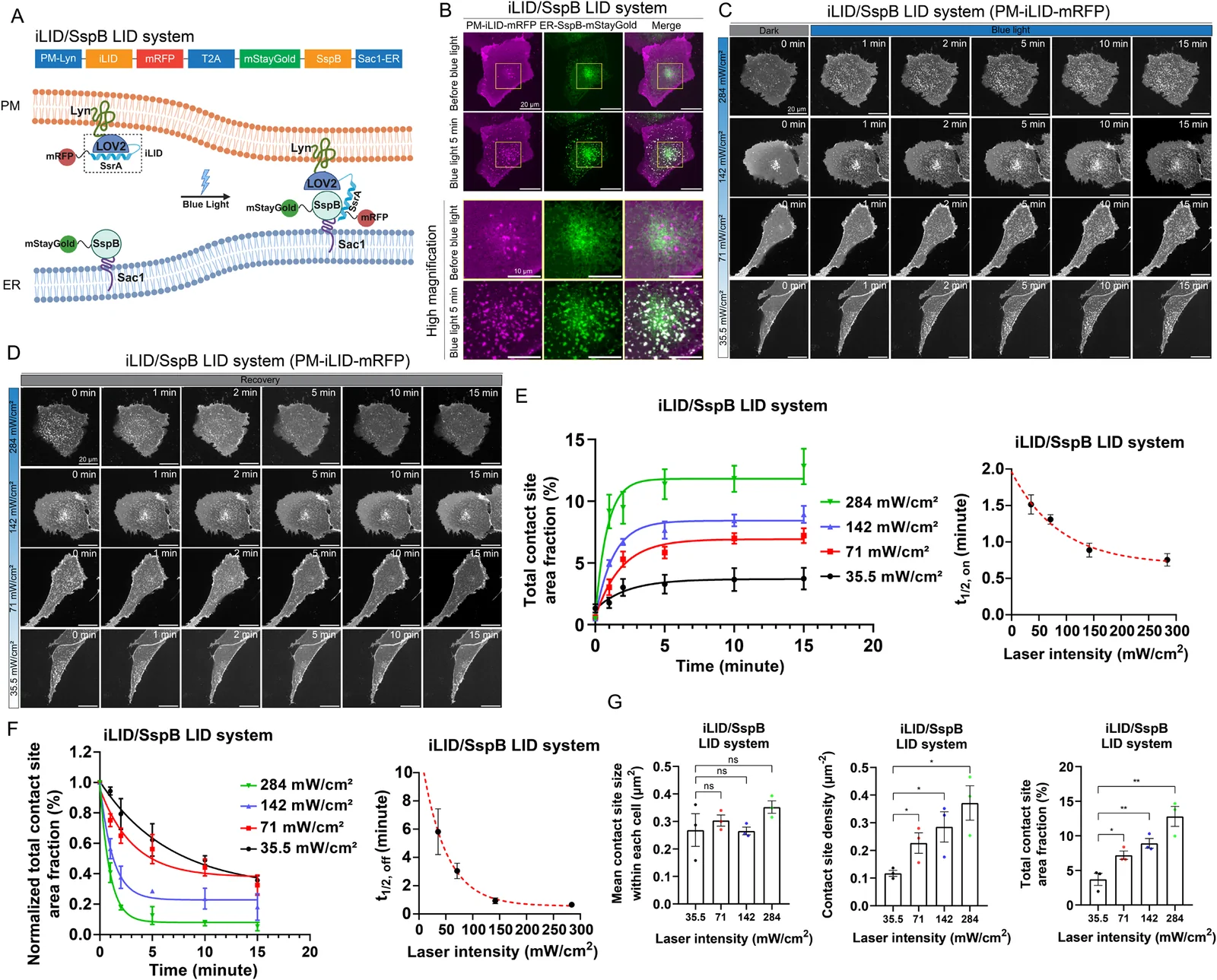
(A) Schematic of construct design and the iLID/SspB LID system. (B) Representative confocal images of U2OS cells before and after blue light stimulation. (C, D) Time-course, intensity-dependent formation, and recovery of blue light-induced ER–PM contact sites. (E, F) Quantification of the total contact-site area fraction and derived half-times *t*_1/2,on_ and *t*_1/2,off_ using single-exponential association and dissociation models. (G) Quantification of contact-site size, density, and area fraction at varying light intensities. Data represent mean ± s.e.m. (*n* = 3). Scale bars: 20 μm (original view) and 10 μm (magnified view). Statistical analysis was performed as described in Figure 1.

To validate whether the iLID/SspB system can effectively induce ER–PM contact-site formation, we transfected cells with the construct PM-iLID-mRFP-T2A-mStayGold-SspB-ER and monitored contact-site formation using confocal microscopy before and after light stimulation. In the dark, PM-iLID-mRFP fluorescence was uniformly distributed along the PM, whereas the SspB-mStayGold-ER exhibited an ER network pattern, with minimal background puncta formation, closely resembling the localization observed for the ABA-inducible CID system. Upon blue-light illumination (470 nm at 20% laser output, corresponding to an intensity of ∼142 mW/cm^2^ at the sample plane), both PM-iLID-mRFP and SspB-mStayGold-ER rapidly reorganized into discrete punctate structures at the cell surface, where they displayed tight colocalization. These punctate ER–PM contact sites formed rapidly in a time-dependent manner, reaching a near-steady state within 5 min, after which the puncta remained largely stable with minimal lateral expansion (Figure 2B). These results demonstrate that our designed light-inducible LID system enables robust and rapid induction and visualization of ER–PM contact sites. As observed in the chemically inducible systems, a small fraction of cells (∼10%) in the iLID/SspB system also exhibited preinduction intracellular punctate signal enrichment in addition to plasma membrane localization (Figure S4A). These intracellular puncta did not colocalize with MAPPER, indicating that they do not represent pre-existing ER–PM contact sites (Figure S4B) and can be excluded from quantitative analysis.

Inspired by our earlier findings that ER–PM contact-site formation can be precisely tuned by ABA or rapamycin concentration and that ABA-induced contacts are reversible, we next tested whether light intensity could similarly control the formation and dissociation kinetics of our light-inducible ER–PM contact system. Increasing 470 nm laser intensity from 5% (∼35.5 mW/cm^2^) to 40% (∼284 mW/cm^2^) progressively accelerated contact-site formation and increased the steady-state contact-site area fraction (Figure 2C). Following 15 min of illumination at each tested laser intensity, withdrawal of blue light triggered rapid dissociation of ER–PM contact sites across all conditions, demonstrating robust reversibility (Figure 2D). Notably, higher illumination intensities resulted in faster dissociation upon light withdrawal. To quantify these effects, the time-dependent changes in total contact-site area fraction during light-induced formation and dark-state dissociation were fitted with single-phase exponential association and dissociation functions, respectively (Figure 2E,F), yielding the induction and recovery half-times (*t*_1/2,on_ and *t*_1/2,off_). Both *t*_1/2,on_ and *t*_1/2,off_ decreased exponentially with increasing 470 nm laser intensity (Figure 2E,F), indicating that light intensity provides precise, bidirectional control over ER–PM contact-site kinetics. The coupled acceleration of both formation and dissociation at higher light intensities suggests a shared kinetic origin. Increased illumination rapidly drives a larger fraction of iLID molecules into the binding-competent conformation, enhancing the effective association rate and promoting rapid contact-site nucleation. Upon light withdrawal, these activated iLID molecules undergo photophysical relaxation to the dark, non-binding state, leading to a rapid loss of productive interactions and accelerated contact-site disassembly. This behavior is consistent with mass-action effects and a nucleation-dominated assembly mechanism, in which light intensity modulates the population dynamics of the activated iLID state rather than altering intrinsic binding affinity. Compared with the ABA-inducible CID system, the iLID/SspB-based LID system exhibited markedly faster dynamics, with rapid induction (5% laser output, *t*_1/2,on_ = 1.51 ± 0.13 min) and near-complete recovery within minutes (5% laser output, *t*_1/2,off_ = 5.82 ± 1.62 min). In contrast, the ABA system required ∼1 h for contact-site dissociation. Together, these results demonstrate the superior temporal reversibility of the iLID/SspB-based ER–PM contact site forming system, enabling repeated cycles of contact formation and dissociation with precisely tunable kinetics controlled by light intensity.

Next, we examined how steady-state ER–PM contact site geometry responds to optical input in the iLID/SspB system. Cells were subjected to increasing 470 nm laser intensities, and ER–PM contacts were allowed to reach steady state after 15 min of continuous illumination. As in the ABA-inducible system, we quantified three geometric parameters: the mean size of contact sites, contact-site density, and the total contact-site area fraction per cell. Increasing blue laser intensity led to a monotonic increase in both contact-site density and total contact-site area fraction, whereas the mean contact-site size remained largely unchanged across the tested intensity range (Figure 2G). This behavior mirrors the inducer dose dependence observed for the ABA system, indicating that the optical input primarily regulates ER–PM contact-site nucleation and overall abundance. In contrast, the lateral dimensions of contact sites appear to be constrained by the intrinsic tether architecture rather than by the strength of the inducing stimulus.

It is worth noting that prior to this work, a light-inducible ER–PM contact site-forming system, termed LiMETER, had been reported.^16^ However, our iLID/SspB-based LID system offers two key advantages. First, LiMETER consists of a single GFP-tagged component localized to the ER, in which blue-light activation of the LOV2 module exposes a polybasic region that binds PI­(4,5)­P_2_ at the PM to induce ER–PM contacts.^16^ As a result, the LiMETER fluorescence signal largely follows the tubular and sheet-like ER network, making it difficult to visually distinguish discrete ER–PM contact site puncta from the surrounding ER structure. In our hands, this leads to a higher baseline puncta count in the absence of light stimulation due to the ER-associated fluorescence distribution, thereby reducing the dynamic range between dark and illuminated states in quantitative analysis (Figure S5A–C). Second, LiMETER relies on light-induced exposure of a polybasic motif that directly binds PI­(4,5)­P_2_, rendering ER–PM contact formation dependent on the local phosphoinositide environment and potentially perturbing endogenous PI(4)­P/PI­(4,5)­P_2_ dynamics at the contact sites. Such lipid-dependent membrane association may complicate studies aimed at dissecting the interplay between ER–PM contacts and lipid signaling. By contrast, our iLID/SspB system operates through lipid-independent protein–protein interactions, minimizing interference with native phosphoinositide signaling. This design enables robust, tunable, and reversible ER–PM contact formation while preserving the physiological lipid landscape, providing a versatile optogenetic platform for precise spatiotemporal manipulation and visualization of ER–PM contacts.

### Systematic Linker-Length Variation Enables Control of ER–PM Contact-Site Stability and Geometry

Emerging evidence suggests that not only the presence but also the density, size, and nanoscale organization of ER–PM contact sites critically modulate local signaling and global cellular behavior.^11,13,14^ For example, in migrating cells, ER–PM contacts display a pronounced front-back gradient in both density and size, with small, highly dynamic contacts at the leading edge and larger, more stable contacts at the trailing edge.^11^ These observations highlight the functional importance of the ER–PM contact site geometry and dynamics. Motivated by this, we sought to leverage the chemically induced dimerization (CID) and LID systems developed here to introduce precise control over the average size of induced ER–PM contact sites as well as the integrated total ER–PM contact area per cell. Such tunability provides a powerful platform for dissecting geometry-dependent functions of ER–PM contacts across diverse cellular contexts, including polarized migration, signal activation, and mechanical remodeling, and offers a generalizable strategy for probing structure–function relationships at MCSs with high spatial and temporal precision.

To achieve tunable control over ER–PM contact site geometry, including average size and total contact area per cell, we first tested whether varying the ER–PM intermembrane spacing at induced contact sites could modulate these geometry parameters. Specifically, we engineered constructs with variable intermembrane spacer lengths by modifying both the ABA-inducible (Figure 3A) and iLID/SspB-based light-inducible (Figure 4A) systems described above. In these designs, rigid α-helical spacers composed of repeated (EAAAR)_n_ motifs^37^ (*n* = 0, 4, 9) were inserted between the membrane-targeting sequences and the dimerization modules (i.e., between the ER- or PM-localization sequence and the ABI or PYL component in the ABA-based system, and between the localization sequence and the iLID or SspB component in the light-inducible system). (EAAAR)_n_ peptides serve as model helices whose helicity is markedly superior to many rationally designed α-helical peptides.^38,39^ Owing to their compact, stable, and highly directional α-helical structure, these peptides have been successfully applied in high-throughput screening of rapamycin-mediated FKBP12-FRB interactions.^40^ Based on previously established length estimates for (EAAAR)_n_ α-helical spacers, in which each (EAAAR) repeat approximately 0.75 nm in length,^37,41^ and previously established length estimates for the dimerization modules,^17,41^ we estimated that our three designed constructs (referred to as (EAAAR)_0_, (EAAAR)_4_, and (EAAAR)_9_ hereafter) produce ER–PM intermembrane spacings of approximately ∼8–10 nm, ∼14–16 nm, and ∼22–24 nm, respectively. Together, these spacings effectively cover the full range of distances (∼10–30 nm) reported for native ER–PM contact sites.^42^

**Figure 3:**
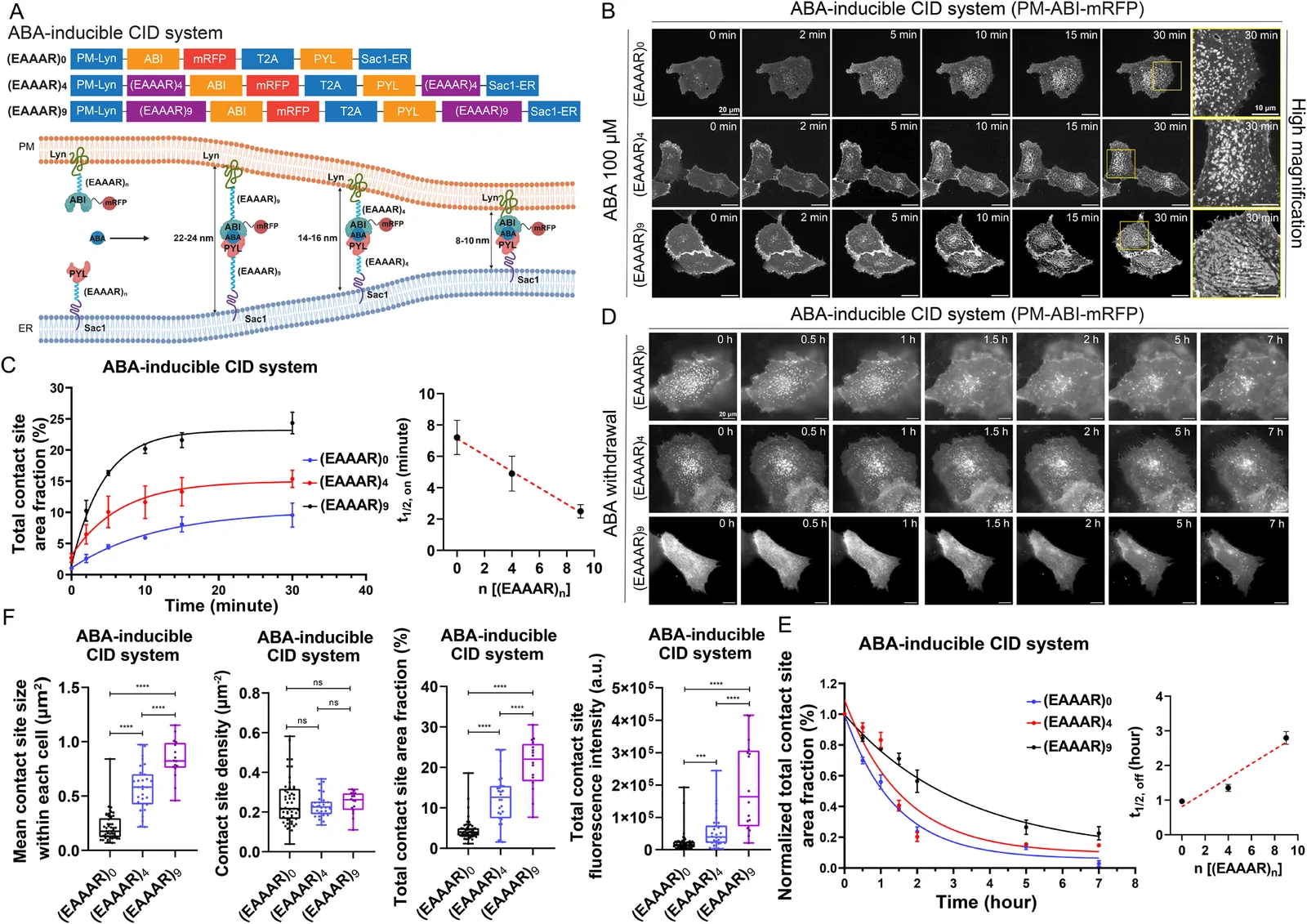
(A) Schematic of constructs and ABA-inducible CID systems with variable spacer lengths to modulate the ER–PM intermembrane distance. (B) Representative time-course and spacer-dependent formation of ABA-induced ER–PM contact sites in U2OS cells. (C) Quantification of the total contact-site area fraction and derived half-time *t*_1/2,on_ using a single-exponential association model. (D, E) Time-course and quantification of contact site dissociation after ABA withdrawal, with *t*_1/2,off_ obtained from a single-exponential decay model. (F) Quantification of contact-site size, density, area fraction, and fluorescence intensity across spacer variants. Data represent mean ± s.e.m. Scale bars: 20 μm (original view) and 10 μm (magnified view). Statistical analysis was performed as described in Figure 1.

**Figure 4:**
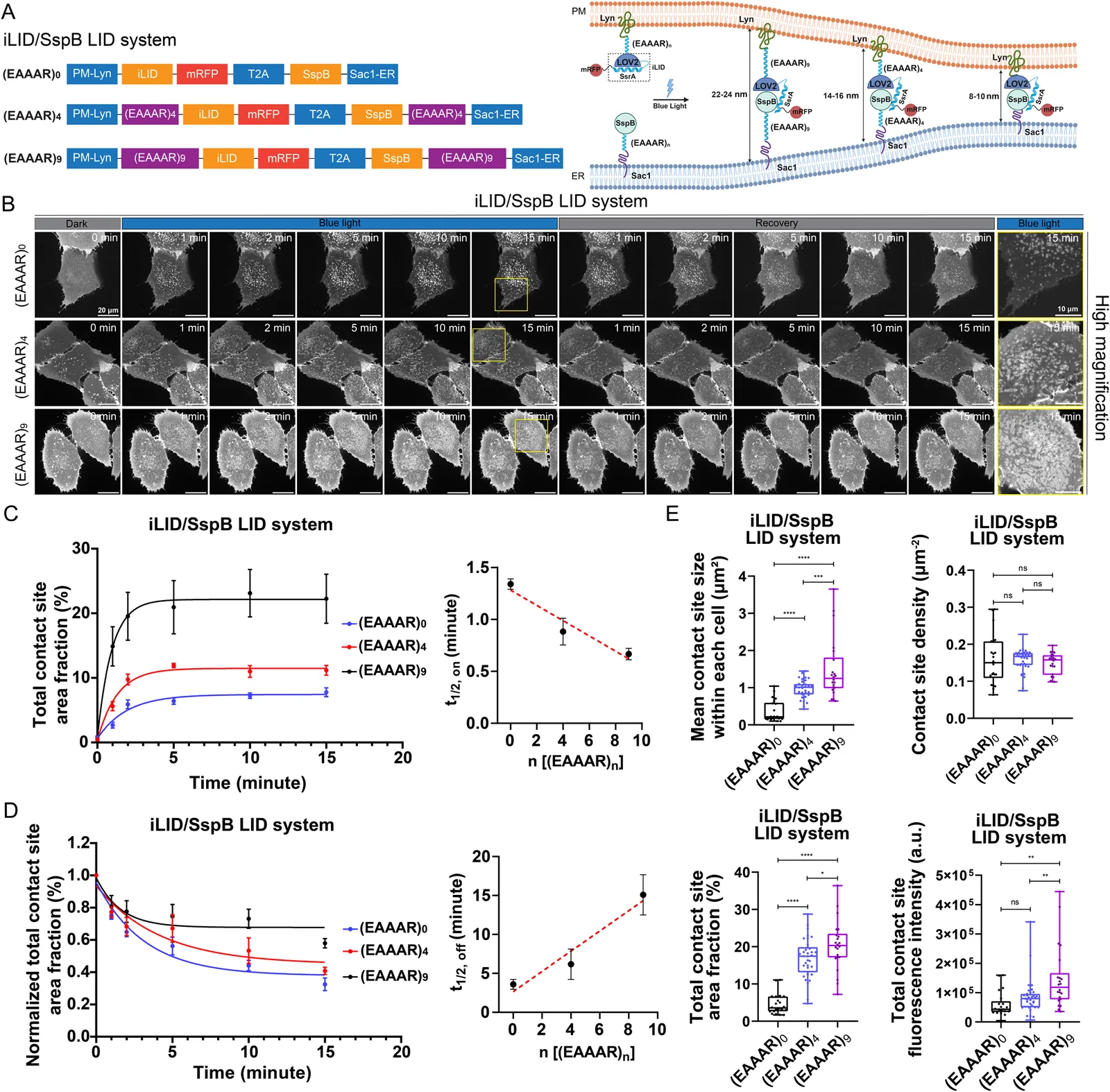
(A) Schematic of constructs and iLID/SspB LID systems with variable spacer lengths to modulate the ER–PM intermembrane distance. (B) Representative time-course of blue light-induced formation and dissociation of ER–PM contact sites in U2OS cells. (C, D) Quantification of the total contact-site area fraction during induction (on) and recovery (off), with derived half-times *t*_1/2,on_ and *t*_1/2,off_ from single-exponential models. (E) Quantification of contact-site size, density, area fraction, and fluorescence intensity across spacer variants. Data represent mean ± s.e.m. Scale bars: 20 μm (original view) and 10 μm (magnified view). Statistical analysis was performed as described in Figure 1.

We first examined whether the engineered (EAAAR)_0_, (EAAAR)_4_, and (EAAAR)_9_ constructs, which differ in linker (i.e., spacer) length, exhibit distinct kinetics of ER–PM contact formation and disassembly. U2OS cells expressing these constructs were subjected to time-course imaging following induction with 100 μM ABA (for the ABA-inducible CID system) or 10% blue-light illumination (for the light-inducible iLID/SspB system; 10% laser output corresponding to ∼71 mW/cm^2^ at the sample plane). In both systems, all three constructs exhibited robust, time-dependent ER–PM contact-site formation (Figures 3B and 4B). We quantified the total ER–PM contact-site area fraction per cell as a function of time after stimulation. Fitting the induction time traces with a single-exponential association model yielded half-times of contact formation (*t*_1/2,on_), which decreased linearly with increasing linker length (Figures 3C and 4C). We next assessed the disassembly kinetics of the three linker variants by washing out ABA (for the ABA-inducible CID system) or terminating blue-light illumination (for the light-inducible iLID/SspB system) (Figures 3D and 4B). Recovery time traces were fit using a single-phase exponential dissociation model to extract the half-times of recovery (*t*_1/2,off_). In contrast to the formation kinetics, *t*_1/2,off_ increased with linker length in both systems (Figures 3E and 4D). Notably, for both the optogenetic and ABA-inducible systems, longer linker constructs exhibited higher residual plateaus, corresponding to incomplete recovery within the observation time window and consistent with the emergence of more persistent contact populations. These observations suggest that linker architecture influences ER–PM contact-site stability and reversibility across both systems. In addition, the optogenetic system displayed higher residual plateaus compared to the ABA-inducible system, indicating more incomplete recovery and greater persistence of induced contacts. Longer linkers may promote larger contact footprints and increased local tether engagement, which could enhance contact persistence and slow recovery. Rapid optogenetic induction may further facilitate membrane reorganization or lateral clustering processes that stabilize induced contacts. Differences in induction and dissociation kinetics between chemical and optogenetic systems may therefore contribute to distinct stabilization behaviors during contact formation. However, the molecular mechanisms underlying these effects remain to be determined.

It is important to note that, in contrast to tuning ABA concentration or iLID/SspB light power, which accelerates both contact formation (decreasing *t*_1/2,on_) and disassembly (decreasing *t*_1/2,off_) (Figures 1E and 2E,F), linker-length modulation accelerates formation but increases *t*_1/2,off_, resulting in prolonged ER–PM contact-site lifetime (i.e., persistence) and increased stability of contact sites. This difference arises because ABA or light intensity controls the fraction of active tethers, enhancing nucleation and turnover without changing intrinsic tether avidity, whereas the linker length alters the physical reach and multivalency of tether interactions, directly influencing contact stability.

Next, we investigated whether varying linker length could systematically tune ER–PM contact site geometry, as predicted. Cells expressing (EAAAR)_0_, (EAAAR)_4_, and (EAAAR)_9_ constructs were stimulated with 100 μM ABA for 15 min (ABA-inducible CID system) or 10% (∼71 mW/cm^2^) blue-light illumination for 5 min (for the light-inducible iLID/SspB system), followed by cell fixation, confocal imaging, and quantitative analysis. In both systems, the mean size, total area fraction, and total fluorescence intensity of induced ER–PM contact sites per cell increased monotonically with linker length, whereas contact-site density remained largely unchanged (Figures 3F and 4E). Consistent with these quantitative trends, cells expressing the long-linker (EAAAR)_9_ variant exhibited large, island-like contact sites in contrast to the discrete small puncta observed for the (EAAAR)_0_ construct (Figures 3B and 4B). These results demonstrate that linker elongation provides a precise and robust means to tune the average size of ER–PM contact sites without substantially altering contact-site density, thereby increasing the total ER–PM contact area per cell across both chemically and optically inducible tethering platforms. Notably, linker-length-dependent tuning selectively regulates contact-site stability and lateral growth, in contrast to inducer-dose-dependent regulation (ABA concentration or laser intensity), which predominantly controls contact-site nucleation and abundance rather than the lateral growth of contacts.

Collectively, these results establish linker length as a tunable design parameter that couples molecular architecture to ER–PM contact site stability and geometry. By systematically tuning the linker length, we can independently modulate contact-site stability, steady-state size, and total contact area without substantially altering contact density. This reveals that the physical reach of inducible tethers directly governs the balance between kinetically labile and avidity-stabilized contact states. Such tunable behavior defines explicit structure–function relationships for MCSs and enables rational engineering of contacts with predefined stabilities and geometries.

### Systematic Tether-Abundance Variation Enables Orthogonal Control of ER–PM Contact-Site Geometry

In addition to varying the linker length (i.e., intermembrane distance), modulating the concentrations of the tethering components on the ER and PM membranes may provide an alternative and orthogonal means of tuning ER–PM contact site geometry. This is because the local density of membrane-anchored tethers directly determines the probability of productive tether–tether encounters, the extent of lateral recruitment following nucleation, and the degree of cooperative stabilization within a contact zone. Consequently, altering tether abundance is expected to influence key geometric parameters of ER–PM contacts without changing the intrinsic intermembrane spacing imposed by the tether (i.e., linker) length.

To test this hypothesis, we selected the long-linker (EAAAR)_9_ construct and subcloned it into a previously established XLone backbone containing a doxycycline (Dox)-inducible promoter^43^ for both the ABA-inducible CID system (hereafter referred to as XLone-ABA) and light-inducible iLID/SspB system (hereafter referred to as XLone-iLID/SspB) (Figure 5A). The XLone system combines a Tet-On 3G promoter and PiggyBac transposon elements, enabling stable genomic integration and tightly regulated, Dox-dependent expression.^43^ This configuration minimizes transgene silencing and supports reversible, tunable control of gene expression, making it particularly suitable for precisely adjusting the expression levels of tethering components on ER and PM membranes.

**Figure 5:**
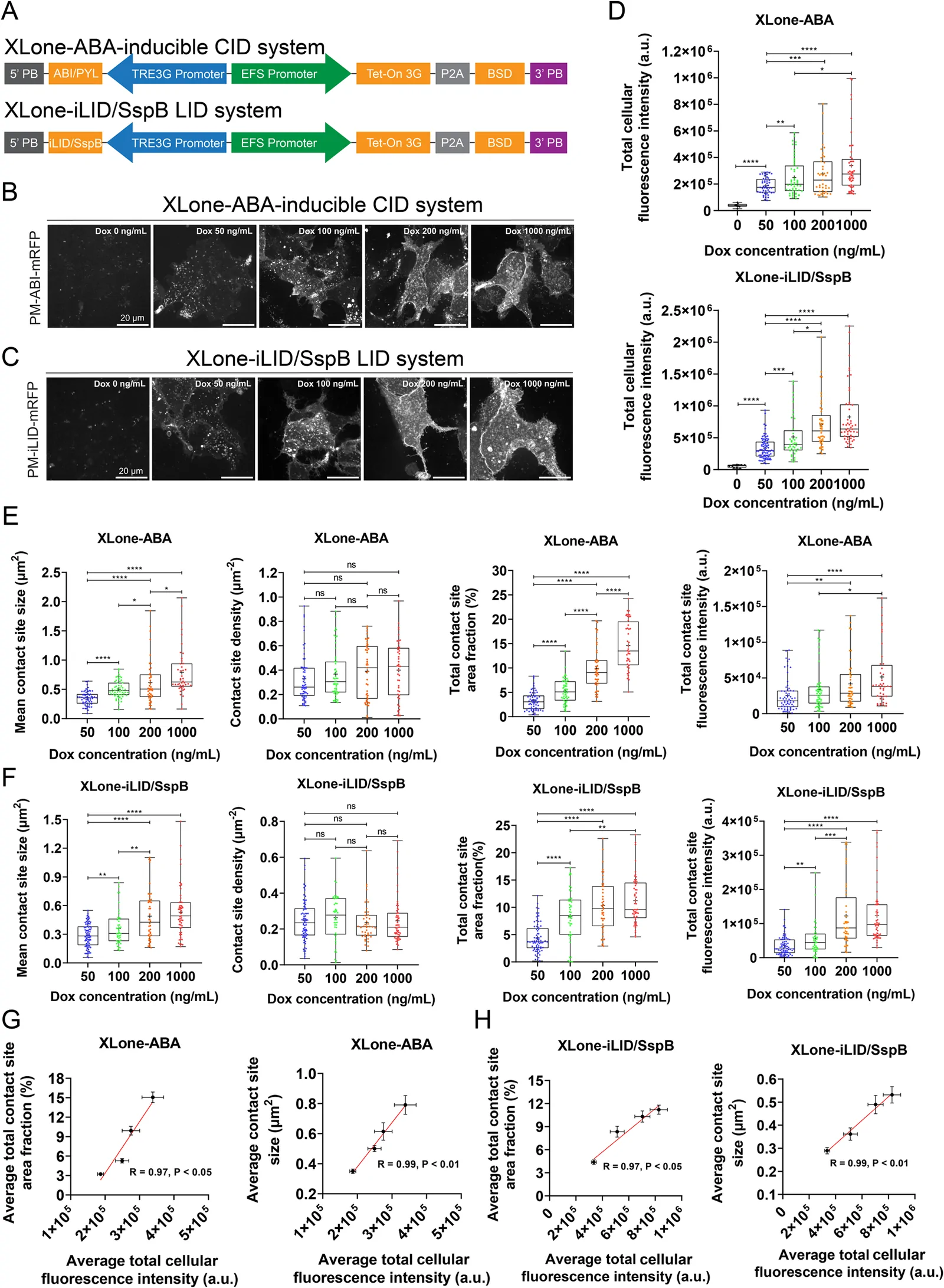
(A) Schematic of XLone-based ABA-inducible CID and iLID/SspB LID systems. (B, C) Representative confocal images of ER–PM contact sites in HEK293T cells with doxycycline-tunable expression followed by chemical or light stimulation. (D) Quantification of total cellular fluorescence intensity as a function of induction level. (E, F) Quantification of contact-site area fraction, size, density, and fluorescence intensity across induction levels for ABA-inducible CID and iLID/SspB LID systems. (G, H) Correlation of total cellular expression with contact-site area fraction and size, with Pearson correlation analysis. Data represent mean ± s.e.m. from three independent biological replicates. Scale bars: 20 μm. Statistical analysis was performed as described in Figure 1.

We transfected cells with the XLone-ABA and XLone-iLID/SspB constructs. 24 h after transfection, the cells were treated with a graded series of Dox concentrations (50, 100, 200, and 1000 ng/mL) to induce distinct expression levels of the tethering components on the ER and PM membranes. After an additional 24 h of Dox induction, the cells were stimulated with 100 μM ABA for 30 min (ABA-inducible CID system) or ∼4 mW/cm^2^ blue-light illumination for 15 min (for the light-inducible iLID/SspB system), followed by cell fixation, confocal imaging, and quantitative analysis. In both systems, mRFP fluorescence intensity, which reports the expression level of the PM-localized tether (PM-ABIcs-mRFP for ABA-inducible CID system or PM-iLID-mRFP for light-inducible iLID/SspB system), increased monotonically with Dox concentration (Figure 5B–D). Notably, although caution should be exercised regarding potential overexpression artifacts, we found that even at the highest Dox concentration tested (1000 ng/mL), the overall expression levels of both XLone-ABA and XLone-iLID/SspB constructs remained substantially lower than those achieved using conventional CMV-promoter-driven expression systems widely used in the field^15,17^ (Figure S6A). Concomitantly, multiple geometry-related parameters of induced ER–PM contact sites, including the mean contact-site size, total contact-site area fraction, and total contact-site fluorescence intensity per cell, increased in a Dox-dependent manner, whereas contact-site density remained largely unchanged (Figure 5E,F), consistent with the linker-length-dependent tuning we observed. Population-averaged analysis further revealed linear relationships between (i) average contact-site size and average PM-tether expression level, (ii) average contact-site density and average PM-tether expression level, (iii) average total contact-site area fraction and average PM-tether expression level, and (iv) average total contact-site fluorescence intensity and average PM-tether expression level (average PM-tether expression was quantified by total cellular mRFP fluorescence; Figures 5G,H and S6B,C). These results demonstrate that tuning the expression level of membrane-anchored tethering components via Dox induction provides a robust and tunable means to modulate ER–PM contact-site geometry, like tuning the linker length.

Together, these results establish tether concentration as an independent and orthogonal design parameter for controlling ER–PM contact-site geometry. Linker length primarily tunes intermembrane spacing and contact stability, while tether abundance modulates the number of engaged tethers per cell; both parameters similarly regulate the size and total area of ER–PM contact sites without substantially altering contact-site density. This framework enables independent modulation of intermembrane spacing and tether density, providing a versatile approach to dissect how the abundance and spatial organization of membrane tethers govern the structural and functional outputs of ER–PM contact sites.

### Induced ER–PM Contact Sites Recapitulate Endogenous Protein Recruitment in a Spacing-Dependent Manner

Thus far, the inducible ER–PM contact-forming systems developed here enable robust and tunable control of membrane apposition; however, it remains unclear whether these induced ER–PM contact sites can recruit endogenous ER–PM contact site-resident proteins or whether they merely represent geometrically enforced membrane proximity without physiological relevance. To address this question, we examined the recruitment of three representative ER–PM contact site-associated proteins to contact sites generated by our ABA-inducible CID and iLID/SspB-based LID systems: (1) the widely used ER–PM contact marker MAPPER,^15^ which faithfully labels physiologically relevant ER–PM contact sites; (2) the voltage-gated potassium channel Kv2.1, a plasma membrane-resident protein that is constitutively enriched at ER–PM contacts through interactions with ER-resident VAP proteins;^44^ and (3) the ER-resident Ca^2+^ sensor STIM1,^9,45^ which translocates to ER–PM contact sites to facilitate Ca^2+^ influx upon ER Ca^2+^ depletion induced by thapsigargin (TG), an inhibitor of SERCA pumps.

To determine whether MAPPER is present at induced contact sites, we established a cell line stably expressing mEGFP-tagged MAPPER and transfected these cells with either the ABA-inducible system or the iLID/SspB-based light-inducible system containing different linker lengths ((EAAAR)_0_, (EAAAR)_4_, and (EAAAR)_9_). Confocal imaging revealed that, in both systems, induced ER–PM contact sites labeled by PM-ABIcs-mRFP (or PM-iLID-mRFP) exhibited strong colocalization with MAPPER across all linker lengths tested, with quantified colocalization fractions exceeding 92% (Figure 6A,B,E). These results indicate that the induced contact sites are not merely geometrically closed ER–PM appositions but instead spatially overlap with endogenous ER–PM contact regions and can recruit established ER–PM contact site markers.

**Figure 6:**
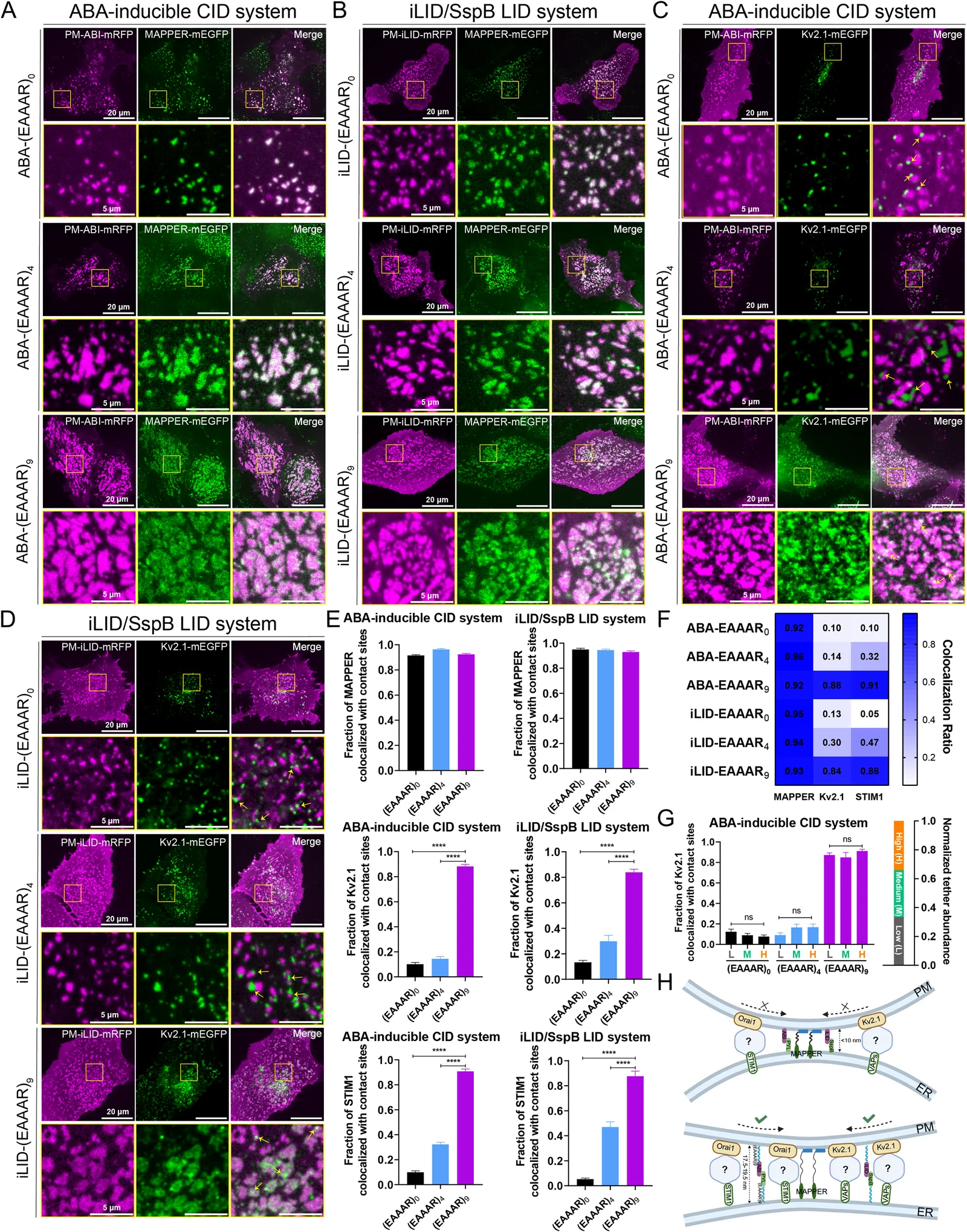
(A, B) Representative confocal images of U2OS cells expressing inducible ER–PM contact systems with varying spacer lengths, showing colocalization with MAPPER following chemical or light stimulation. (C, D) Corresponding analysis of Kv2.1 localization relative to inducible contact sites. (E) Quantification of the fraction of MAPPER, Kv2.1, and STIM1 colocalized with inducible contact sites across spacer variants in both CID and LID systems. Statistical analysis was performed as described in Figure 1. (F) Heatmap summarizing colocalization trends. (G) Kv2.1 colocalization as a function of tether abundance within each spacer group in the ABA-inducible CID system. Statistical comparisons among groups within the same spacer length were performed using one-way ANOVA. (H) Schematic representation of intermembrane distance manipulation and the corresponding spatial requirements for MAPPER, Kv2.1, and STIM1. Data represent mean ± s.e.m. from three independent experiments. Scale bars: 20 μm (original view) and 5 μm (magnified view).

We next examined whether the endogenous ER–PM contact site-resident protein Kv2.1 is recruited to the induced contact sites. mEGFP-tagged Kv2.1 was coexpressed with constructs containing different linker lengths. In both ABA- and iLID/SspB-based systems, Kv2.1 recruitment was strongly dependent on linker length. For short-linker constructs (EAAAR)_0_ and (EAAAR)_4_, induced ER–PM contact sites were formed preferentially adjacent to pre-existing Kv2.1-positive puncta, but these Kv2.1 clusters were largely excluded from the induced contact regions (Figure 6C,D). In contrast, for the long-linker (EAAAR)_9_ construct, induced ER–PM contact sites showed substantial colocalization with Kv2.1, with quantified colocalization fractions exceeding 84% (Figure 6C–E). These observations suggest that Kv2.1-containing complexes are excluded from narrow ER–PM gaps (<∼16 nm) but can be largely accommodated when the intermembrane spacing increases to ∼22–24 nm. Although Kv2.1 is known to interact with ER-resident VAP proteins, the size of these individual components alone is insufficient to fully account for the observed exclusion under short-linker conditions. Previous studies have shown that Kv2.1 organizes specialized ER–PM junctions by recruiting additional ion channels and coupling them into higher-order signaling complexes.^46,47^ Our results therefore raise the possibility that Kv2.1-associated ER–PM contact sites comprise larger multiprotein assemblies that require increased intermembrane spacing for stable incorporation.

We further examined whether STIM1 is recruited to inducible ER–PM contact sites during the SOCE. Cells coexpressing STIM1–mEGFP and the inducible tethering systems were first used to establish ER–PM contact sites, after which ER Ca^2+^ depletion was induced by TG treatment. Prior to TG addition, STIM1–mEGFP displayed a typical ER-like distribution, which remained largely unchanged upon initial contact site induction. Following TG treatment, STIM1 rapidly reorganized into characteristic puncta corresponding to ER–PM junctions. Similar to Kv2.1, STIM1 recruitment exhibited a clear dependence on the intermembrane spacing. In cells expressing the short-linker ((EAAAR)_0_) construct, STIM1 puncta were adjacent to but largely excluded from the induced contact sites in both the ABA and iLID/SspB systems. In cells expressing the intermediate linker ((EAAAR)_4_), partial recruitment of STIM1 to induced contact sites was observed, with colocalization fractions consistently higher than those measured for Kv2.1 (Figures S7 and 6E,F). Notably, STIM1 puncta appeared to locally reorganize or compress the preformed induced contact structures. In contrast, in cells expressing the long-linker ((EAAAR)_9_) construct, TG-induced STIM1 puncta were efficiently recruited to the induced ER–PM contact sites, with colocalization fractions exceeding 88%, without disrupting contact-site morphology (Figures S6 and 6E,F). These findings indicate that STIM1 recruitment, like that of Kv2.1, requires a minimum ER–PM intermembrane spacing, consistent with previous reports on the spatial requirements for STIM1–Orai1 coupling during SOCE.^17^ Notably, the recruitment efficiencies observed for STIM1 at an intermediate linker (EAAAR)_4_ are higher than those for Kv2.1, suggesting that different ER–PM contact site-resident proteins have distinct spatial thresholds for incorporation, likely reflecting differences in their molecular architecture and interaction networks.

Having established that linker length (i.e., intermembrane spacing) influences the recruitment of ER–PM contact site-resident proteins, we next examined whether tether abundance exerts a similar effect. Cells expressing the ABA-inducible system were stratified into low-, medium-, and high-tether-abundance groups within each linker condition, and Kv2.1 localization relative to induced contact sites was quantified. No significant differences were observed among the different abundance groups within the same linker condition (Figure 6G), indicating that tether abundance does not measurably impact the recruitment of ER–PM contact site-resident proteins.

Taken together, these results demonstrate that both ABA- and iLID/SspB-based inducible systems generate ER–PM contact sites that are competent to recruit endogenous ER–PM contact site proteins and that this recruitment is primarily governed by intermembrane spacing dictated by linker length, rather than by tether abundance (Figure 6H). Importantly, although both linker length and tether abundance can modulate mean contact-site size, only linker length, unlike tether abundance, provides additional control over the recruitment of ER–PM contact-site-resident proteins. Together with inducer dose (ABA concentration or light intensity), which primarily controls contact-site density without altering contact-site size, these results establish three orthogonal control parameters, namely, inducer dose, linker length, and tether abundance, that independently regulate ER–PM contact-site density, size, and recruitment of ER–PM contact site-resident proteins. This integrated framework provides a versatile platform for systematically probing how ER–PM contact site geometry influences protein recruitment and, more broadly, functional outcomes. Beyond their applications in mammalian cells, the overall design principles of these inducible ER–PM tethering platforms may be adaptable to other model organisms, including budding yeast, which has long served as a powerful system for studying MCSs. Such future adaptations would likely require optimization of membrane-targeting sequences, linker architecture, codon usage, and expression levels to accommodate differences in membrane composition, cellular geometry, and endogenous ER–PM contact site organization between yeast and mammalian cells.

## Conclusions

We have developed two complementary and independent tunable platforms for precise, reversible, and systematic modulation of ER–PM contact sites, combined with high-contrast visualization in living cells. The ABA-inducible CID system provides nontoxic, reversible chemical control, while the iLID/SspB optogenetic system enables rapid, reversible, light-dependent modulation with superior temporal resolution; both allow dose-dependent regulation of contact-site formation and disassembly kinetics as well as the contact-site density and total area fraction per cell, without substantially changing the mean size of contact sites. Importantly, these perturbations systematically modulate the population-average geometric properties of ER–PM contact sites rather than precisely controlling the geometry or dynamics of individual contact sites, which remain inherently heterogeneous in living cells. In contrast, systematic variation of α-helical linker length or inducible tether expression (i.e., tether abundance) precisely tunes the lateral growth and stability of contact sites, reflected in increased mean size and total contact area, without altering their density. In addition, linker length variation further tunes recruitment of ER–PM contact site-resident proteins, whereas inducible variation of tether abundance does not alter the recruitment of the tested ER–PM contact site-resident proteins. Together, these complementary mechanisms achieve independent and tunable modulation of contact-site dynamics, density, size, and resident protein recruitment, addressing a long-standing limitation in the field where existing approaches typically alter multiple parameters simultaneously. These complementary mechanisms establish predictive relationships between contact-site dynamics, geometry, and molecular recruitment, defining independent parameters to modulate contact-site dynamics, density, size, and resident protein recruitment (Figure 7).

**Figure 7:**
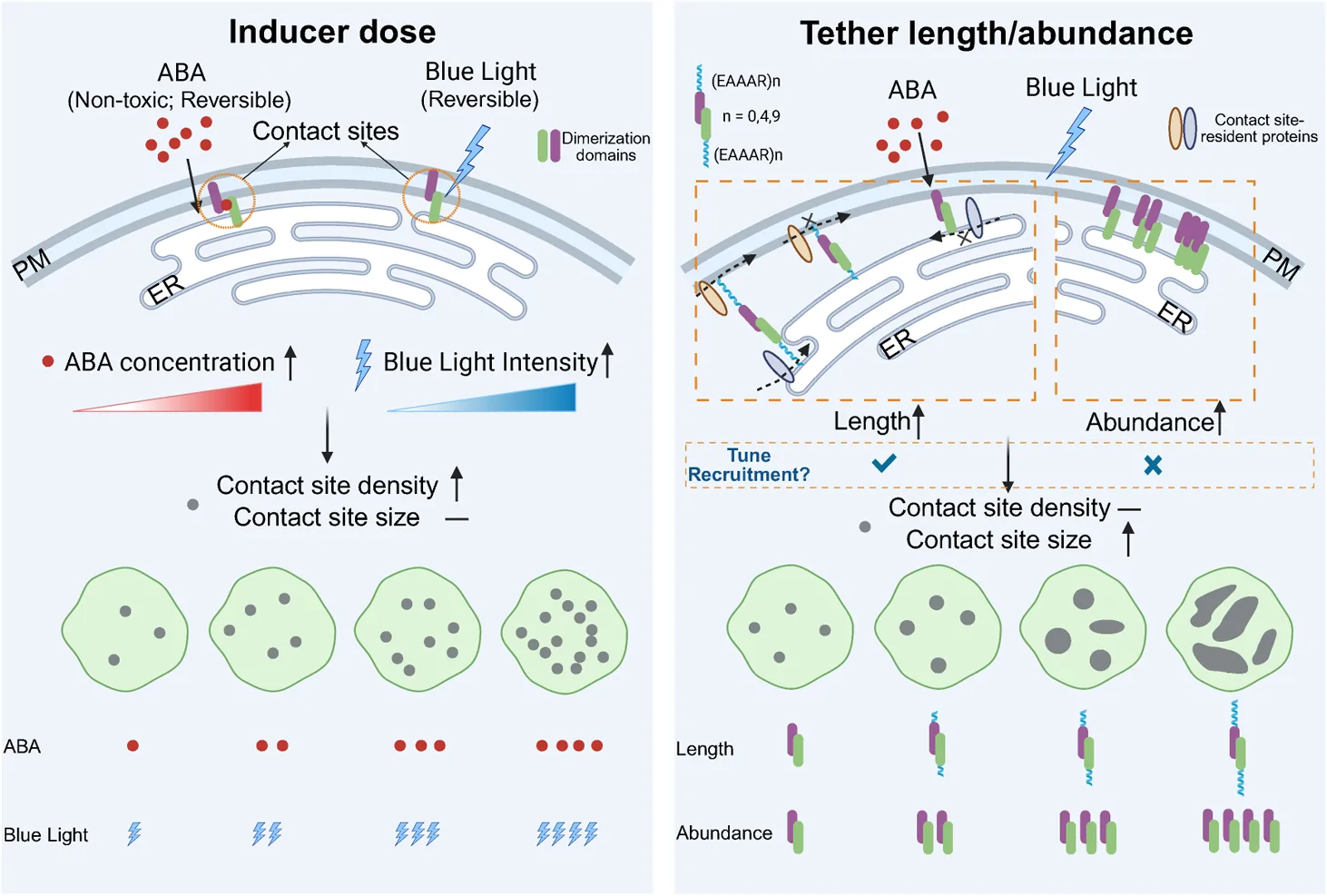
Schematic summary of complementary mechanisms regulating ER–PM contact sites. Schematics were created using BioRender.com.

Beyond providing versatile experimental tools, the ability to systematically modulate population-average contact-site geometry provides an experimental framework for investigating structure–function relationships at ER–PM contact sites. For example, controlled perturbation of contact-site density, size, intermembrane spacing, stability, or molecular composition could be used to examine how these geometric parameters influence downstream cellular processes such as Ca^2+^ signaling, lipid transport, membrane homeostasis, metabolic coordination, and directional cell migration. The tunable and reversible nature of these system perturbation-minimized studies enables repeated induction–recovery cycles and systematic exploration of spatial and temporal regulations. Importantly, the modular design of these platforms is generalizable, providing a blueprint for manipulating other inter-organelle contact sites and expanding the experimental toolkit for studying intracellular organization, signaling compartmentalization, and disease-relevant dysregulation. Altogether, our work establishes a versatile, predictive, and high-fidelity framework for engineering and interrogating MCSs, linking molecular design, geometry, and composition to potential functional outcomes in living cells.

## Supplementary Material



## Data Availability

All data and code necessary to evaluate and reproduce the findings reported in this study are available in the main text, the Supporting Information, and/or the Zenodo repository. The MATLAB codes used for ER–PM contact site analysis and colocalization analysis are publicly available on GitHub at https://github.com/thezhoulab/MATLAB-scripts-for-ER-PM-Contact-Site. The experimental datasets, source data, and plasmid maps used in this study are also available at Zenodo.^48^

## Notes

Detailed experimental materials and methods; and supplementary characterization and validation data, including characterization and quantitative evaluation of rapamycin- and ABA-induced CID systems, subcellular localization analysis of Lyn-mRFP, cell viability analysis of chemical inducers, characterization and comparison of iLID/SspB LID and LiMETER systems, expression-level analysis of inducible systems, and analysis of linker-dependent STIM1 recruitment (PDF)
