# Supplementary material for: Tunable Chemical and Optical Modulation of ER-Plasma Membrane Contact-Site Geometry, Dynamics, and Protein Recruitment with High-Fidelity Visualization
Source: Anal Chem. 2026 Aug 11;98(33):24473–88. doi: 10.1021/acs.analchem.6c03857 (PMC13523554; doi:10.1021/acs.analchem.6c03857)
Supplement: Supplementary file 1 [file ac6c03857_si_001.pdf]

Supporting Information for

Tunable Chemical and Optical Modulation of ER-Plasma Membrane Contact-Site Geometry, Dynamics, and Protein Recruitment with High-Fidelity Visualization

Shaoqing Zhang[1,†], Jinyu Fei[1,†], Yuanmin Zheng[1,2], Ruobo Zhou[1,2,3,4,*]

[1] Department of Chemistry, The Pennsylvania State University, University Park, PA 16802, USA

[2]Department of Biomedical Engineering, The Pennsylvania State University, University Park, PA 16802, USA

[3]Department of Biochemistry and Molecular Biology, The Pennsylvania State University, University Park, PA 16802, USA

[4]The Huck Institutes of Life Sciences, The Pennsylvania State University, University Park, PA 16802, USA

[†]S.Z. and J.F. contributed equally to this work

*To whom correspondence may be addressed. Email: ruobo.zhou@psu.edu

Table of Contents

Materials and Methods S3

1. Plasmid construction S3
2. Cell culture S4
3. Stable cell line generation S4
4. Cell transfection S5
5. ABA and rapamycin induction and withdrawal assays S5
6. Blue light induction and withdrawal assay S6
7. Widefield epi-fluorescence imaging S7
8. Confocal imaging S7
9. MTT cell viability assay S7
10. Quantification of fluorescence images S8
11. Statistical analysis S9

Supplementary Figures S11

Figure S1. Characterization of the rapamycin-inducible CID system and subcellular localization of Lyn-mRFP in CID systems. S11

Figure S2. Cell viability analysis of ABA and rapamycin. S14

Figure S3. Quantitative analysis of the rapamycin-inducible CID system. S14

Figure S4. Subcellular localization of Lyn-iLID-mRFP in iLID/SspB LID system. S16

Figure S5. Comparison of the iLID/SspB LID and LiMETER systems. S17

Figure S6. Expression-level analysis of the XLone-inducible ABA CID or iLID/SspB LID systems. S18

Figure S7. STIM1 recruitment in systems with varying linker lengths. S20

References S22

Materials and Methods

1. Plasmid construction

All plasmids and DNA fragments used in this study were obtained from Addgene or synthesized by commercial providers. Coding sequences were amplified by PCR using high-fidelity DNA polymerase (New England Biolabs, M0491L), and plasmids were assembled using Gibson assembly (New England Biolabs, E5510S) according to the manufacturer's instructions. Unless otherwise noted, constructs were cloned into mammalian expression vectors under the control of a CMV promoter.

For the ER-PM contact-forming chemically induced dimerization (CID) and light-induced dimerization (LID) systems, rapamycin-inducible (FBR-mRFP-T2A-FKBP), abscisic acid (ABA)-inducible (ABIcs-mRFP-T2A-PYLcs), and blue-light inducible (iLID-mRFP-T2A-SspB) expression cassettes were generated. Individual coding sequences were derived from Addgene plasmids (#108137 and #40896; rapamycin system, #134999; ABA system, and #171038; LID system), amplified by PCR, and assembled into a single open reading frame containing a self-cleaving T2A peptide between the two fusion proteins. For PM-targeted constructs, the N-terminal membrane-localized palmitoylation/myristoylation signal of Lyn kinase (MGCIKSKGKDS) was fused in-frame to the N terminus of FRB (rapamycin system), ABIcs (ABA system), or iLID (LID system), via a flexible linker (AGADPTRSANSGAGAGAGAILSR). For ER-targeted constructs, the C-terminal ER localization sequence of human Sac1 phosphatase (residues 521-587) was fused in-frame to the C terminus of FKBP (rapamycin system), PYLcs (ABA system), or SspB (LID system) via a flexible linker (GSGAGAGAGAILNSRV). In all constructs, the PM-targeted and ER-targeted fusion proteins were encoded within a single bicistronic expression cassette and separated by a T2A peptide, enabling stoichiometric expression of the two proteins from a single promoter through ribosomal skipping. To generate variants with altered linker rigidity and length,

synthetic DNA fragments encoding α-helical linkers ($(EAAAR)_4$ or $(EAAAR)_9$; Twist Bioscience) were inserted to the original short linker (*i.e.*, $(EAAAR)_0$) by PCR amplification and Gibson assembly. For mEGFP-MAPPER, mEGFP-Kv2.1, and mEGFP-STIM1 constructs, coding sequences were obtained from Addgene plasmids (#117721, #111532, and #18857, respectively), and mEGFP was inserted in-frame using PCR amplification and Gibson assembly. For XLone-ABA and XLone-iLID/SspB constructs, the constitutive CMV-driven expression cassette was subcloned into the XLone backbone (Addgene, plasmid# 96930) using PCR amplification and Gibson assembly. All plasmids were verified by Sanger DNA sequencing.

2. Cell culture

Human osteosarcoma (U2OS) and human embryonic kidney 293T (HEK293T) cells were maintained in Dulbecco's Modified Eagle Medium (DMEM; Corning, 10-013-CV) supplemented with 10% (v/v) fetal bovine serum (FBS; Gibco, A52567-01) and 1% (v/v) penicillin-streptomycin (Gibco, 10378-016). Cells were maintained at 37 °C in a humidified incubator with 5% CO_2. For routine passaging, cells were dissociated using 0.25% trypsin-EDTA (Corning, 25-053-CI). For imaging experiments, cells were seeded onto 18-mm round glass coverslips (Electron Microscopy Sciences, 72222-01) placed in 12-well tissue culture plates.

3. Stable cell line generation

Stable cell lines were established using the PiggyBac transposon system. To generate cells stably expressing MAPPER, the XLone-MAPPER-mEGFP construct (derived from the Xlone backbone, Addgene plasmid #96930) was used as the transposon vector. Cells seeded in 6-well plates were transfected at approximately 60-80% confluency with 1.6 µg of the transposon plasmid together with 0.4 µg of the PBase transposase plasmid using TransIT-LT1 reagent, following the manufacturer's instructions. 48 h post-transfection, cells were placed under selection with 10

μg/mL Blasticidin. Selection was maintained for 7-14 days, with medium replaced every 2-3 days, until stable cell populations expressing MAPPER-mEGFP were obtained.

4. Cell transfection

Plasmid transfection was performed using TransIT®-LT1 reagent (Mirus Bio, MIR2300) according to the manufacturer's instructions. Briefly, cells at 60-80% confluency were transfected with 1.0 μg of plasmid DNA diluted in 100 μL of Opti-MEM (Gibco, 31985-070). Subsequently, 3 μL of TransIT-LT1 reagent was added to the diluted DNA, gently mixed, and incubated for 15 min at room temperature (RT) to allow DNA-lipid complex formation. The transfection mixture was then added dropwise to cells cultured in complete growth medium. After 6-12 hours, the medium was replaced with fresh DMEM supplemented with 10% FBS to facilitate cell recovery and transgene expression. For experiments using Xlone-based inducible constructs, doxycycline (Sigma, D3447-500mg) at the indicated concentrations was added to the culture medium 48 h after transfection to induce transgene expression. Cells were subjected to live-cell imaging or fixed for downstream analyses 24-48 h post-transfection, depending on the experimental design.

5. ABA and rapamycin induction and withdrawal assays

For live-cell imaging experiments, 100 mM ABA (TCI America, A1698) and 100 mM rapamycin (MilliporeSigma, 553210) stock solutions were diluted to the indicated working concentrations in Gibco™ Live Cell Imaging Solution (LCIS; Thermo Fisher Scientific, A14291DJ). U2OS cells were pre-equilibrated by rinsing once with pre-warmed imaging solution and incubating at 37 °C for at least 15 minutes prior to stimulation. ABA or rapamycin was then added directly to the imaging chamber during image acquisition, and confocal imaging was performed over a 0-30 minute time course following compound addition to monitor ER-PM contact site formation. For withdrawal experiments, cells were treated with ABA or rapamycin for 30 minutes, after which

the compounds were removed by washing the cells four times with pre-warmed LCIS. The cells were then maintained in fresh LCIS, and confocal imaging was performed over a 0-7 hour time course following compound withdrawal to assess the reversibility of ER-PM contact site formation. For fixed-cell assays, ABA stock solutions (100 mM in DMSO) were aliquoted and stored at -20 °C. Before fixation, cells were treated with 100 μM ABA diluted in complete DMEM (with 10% FBS, 1% penicillin-streptomycin) and incubated at 37 °C for 30 minutes. Following induction, cells were immediately washed with phosphate-buffered saline (PBS) and fixed using 4% (w/v) paraformaldehyde containing 4% (w/v) sucrose in PBS for downstream procedures.

6. Blue light induction and withdrawal assay

For live-cell imaging assays, U2OS cells were pre-equilibrated by rinsing once with pre-warmed LCIS and incubating at 37 °C for at least 15 minutes prior to light stimulation. Blue light illumination was applied using a 470-nm laser line (89 North) at 5-40% output power, corresponding to a laser power of 0.022-0.177 mW measured at the sample plane using an optical power meter (ThorLabs, PM130D). Confocal imaging was performed during continuous blue light illumination over a 0-15 minute time course to monitor ER-PM contact site formation. For withdrawal experiments, blue light illumination was terminated after 15 minutes of stimulation. Cells were then maintained in fresh LCIS without blue light exposure, and confocal imaging was performed over a 0-15 minute time course following blue light withdrawal to assess the reversibility of ER-PM contact site formation.

For fixed-cell assays, HEK293T cells expressing XLone-based constructs were stimulated prior to fixation using a custom-built blue light illumination system. The system consisted of a 465-nm blue LED light source powered by a DC power supply, with output light intensity adjusted to the desired level. Cells were exposed to continuous blue light illumination for defined durations. To

minimize unintended activation, cells were shielded from ambient blue light prior to stimulation. Immediately following stimulation, cells were fixed using 4% (w/v) paraformaldehyde containing 4% (w/v) sucrose in PBS for downstream procedures.

7. Widefield epi-fluorescence imaging

Widefield epi-fluorescence imaging was performed using a custom-built microscope based on an Olympus IX-73 body, equipped with a 20× (N.A. 0.80) UPlanXApo air-immersion objective (Olympus), a 60× (N.A. 1.4) PlanApo oil-immersion objective (Olympus), and a CMOS Camera (Thorlabs, CS126MU). A Lumencor SOLA light engine (SOLA U-nIR) was used as the light source for illumination. Unless otherwise indicated, the 60× oil-immersion objective was used to image U2OS cells during rapamycin- and ABA-induced time-course and recovery experiments.

8. Confocal imaging

Confocal imaging was performed using a custom-built microscope based on an Olympus IX-83 inverted microscope body, equipped with a CrestOptics X-Light V3 spinning-disk confocal unit. Illumination was provided by an LDI-NIR-7 Laser Diode Illuminator (89 North). Images were collected using either a 60× (NA 1.42) UPlanXApo or a 100× (NA 1.40) UPlanSApo oil-immersion objective (Olympus) and recorded with an ORCA-Fusion BT digital CMOS camera (Hamamatsu, C15440-20UP). Image acquisition was controlled through the Multi-Dimensional Acquisition module in Micro-Manager 2.0.0, enabling multichannel and z-stack imaging. Z-series were acquired from the apical to basal planes of U2OS cells with a step size of 0.2-0.5 μm. Final confocal images were generated by maximum-intensity projection of the z-stacks.

9. MTT cell viability assay

Cell viability was assessed using the MTT assay following the protocol previously described[1]. Cells were seeded into 96-well plates at a density of 3000 cells per well and treated with the

indicated compounds for 72 h. MTT (MedChemExpress, HY-15924) reagent was then added to each well and incubated for 4 h at 37 °C to allow reduction of MTT to insoluble formazan crystals by metabolically active cells. Formazan crystals were subsequently solubilized, and absorbance was measured using a BioTek ELx808 microplate reader. Relative cell viability was calculated by normalizing absorbance values to vehicle-treated controls. Half-maximal inhibitory concentration (IC_{50}) values were determined by nonlinear regression analysis of dose-response curves using GraphPad Prism 9 (GraphPad Software).

10. Quantification of fluorescence images

Identification and morphological analysis of ER-PM contact site puncta, as well as quantification of mRFP fluorescence intensity, were performed using custom MATLAB scripts. Fluorescence images were converted to single-channel grayscale and normalized to 16-bit intensity. Small punctate structures were enhanced using a morphological top-hat filter, followed by automated thresholding using Otsu's method to generate binary masks. Morphological opening and size filtering were applied to remove noise and small artifacts. Puncta were further refined based on shape parameters, including circularity and eccentricity, to exclude elongated or irregular objects. The resulting masks were applied to the original images to extract puncta signals, and processed images were exported for downstream quantitative analysis.

Total contact site area fraction was calculated as the total area of detected contact site puncta divided by the total cell area for each cell. Average contact site size was defined as the mean area of individual contact site puncta per cell. Contact site density was calculated as the number of detected contact site puncta per cell divided by the total cell area. For withdrawal assays, the normalized total contact sites area fraction at each time point was calculated by dividing the contact sites total area fraction by the corresponding value at time 0. mRFP fluorescence intensity was

quantified as the mean fluorescence intensity across the entire cell. Total contact site fluorescence intensity was calculated as the product of the mean mRFP fluorescence intensity and the total contact site area. Total cellular fluorescence intensity was calculated as the product of the mean mRFP fluorescence intensity and the total cell area. Fractions of MAPPER, Kv2.1, or STIM1 colocalized with inducible ER-PM contact sites were calculated by measuring the area of each protein overlapping with the detected contact site masks within the selected field of views (FOVs) and dividing it by the total area occupied by MAPPER, Kv2.1, or STIM1 within the same FOVs.

To characterize the temporal kinetics of ER-PM contact site formation, total contact site area fraction at each time point after ABA/Rapamycin addition or blue light illumination was fitted using single exponential association model according to **Equation S1**:

$$Y(t) = Y_0 + (Plateau - Y_0)(1 - e^{-kt}) \quad \textbf{(S1)}$$

where Y_0 represents the initial contact-site area fraction at $t = 0$, Plateau denotes the maximal steady-state area reached at long time points, and k is the rate constant, which was used to derive the half-time of induction ($t_{1/2,\mathrm{on}} = \ln 2 / k$).

For the recovery process, time-course data of normalized total contact site area fraction were fitted using single exponential dissociation model according to **Equation S2**:

$$Y(t) = (Y_0 - Plateau)e^{-kt} + Plateau \quad \textbf{(S2)}$$

where Y_0 represents the initial value at the start of recovery (t = 0), Plateau denotes the final steady-state value after complete dissociation, and k represents the rate constant used to calculate the half-time of recovery ($t_{1/2,\mathrm{off}} = \ln 2 / k$).

11. Statistical analysis

All data visualization and statistical analyses were performed using GraphPad Prism 9. Data shown in the figures are presented as mean ± s.e.m. For boxplots, the center line represents the median,

boxes indicate the interquartile range (first to third quartiles), and whiskers denote the minimum and maximum values excluding outliers, and the plus sign (+) marks the mean. Comparisons between two groups were performed using two-tailed unpaired Student's *t* tests unless otherwise specified; comparisons among three groups were analyzed using one-way ANOVA. A p value less than 0.05 was considered statistically significant. In all figures, significance is coded as: ns non-significant, *$P < 0.05$, **$P < 0.01$, ***$P < 0.001$, ****$P < 0.0001$.

Supplementary Figures

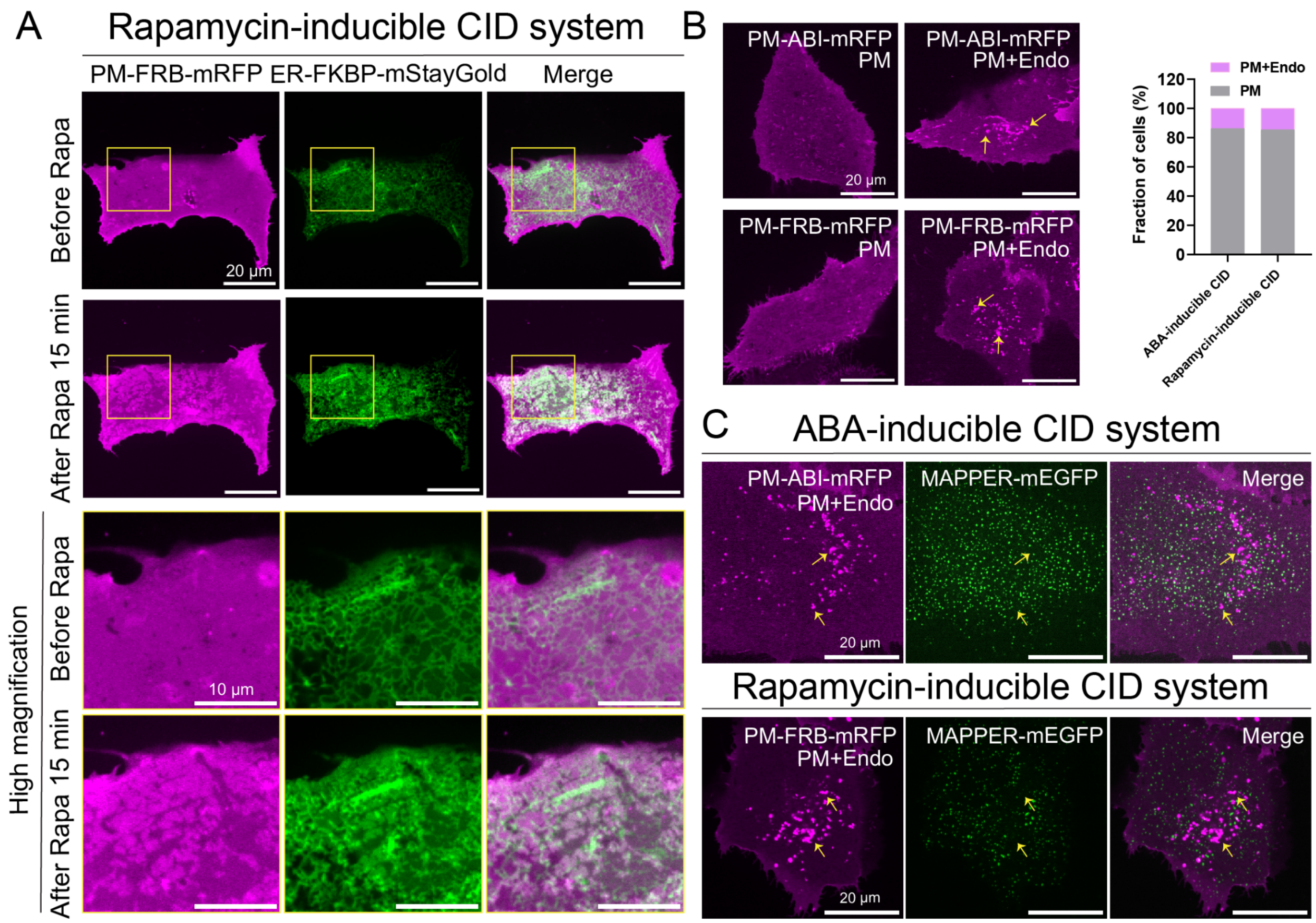

Figure S1. Characterization of the rapamycin-inducible CID system and subcellular localization of Lyn-iLID-mRFP in CID systems. (**A**) Representative confocal images of U2OS cells expressing the rapamycin-inducible construct (PM-FRB-mRFP-T2A-mStayGold-FKBP-ER) before and 15 min after treatment with 100 nM Rapamycin. Yellow boxed regions are shown at higher magnification below. Scale bar: 20 μm (original view); 10 μm (magnified view). (**B**) Left: representative confocal images showing two distinct localization patterns of Lyn-mRFP observed in both the ABA- and rapamycin-inducible CID systems. "PM" indicates that Lyn-iLID-mRFP (i.e., PM-iLID-mRFP) is predominantly localized to the plasma membrane. "PM+Endo" indicates that Lyn-iLID-mRFP is localized to both the plasma membrane and endosomal vesicles. Yellow arrows indicate endosomal vesicles. Scale bar: 20 μm. Right: quantification shows the fraction of U2OS cells exhibiting PM localization or PM+Endo localization across the ABA- and Rapamycin-

inducible systems. (**C**) Representative confocal images of U2OS cells co-expressing either the ABA- or rapamycin-inducible CID system together with MAPPER-mEGFP prior to inducer treatment. Scale bar: 20 μm. Yellow arrows indicate endosomal vesicles.

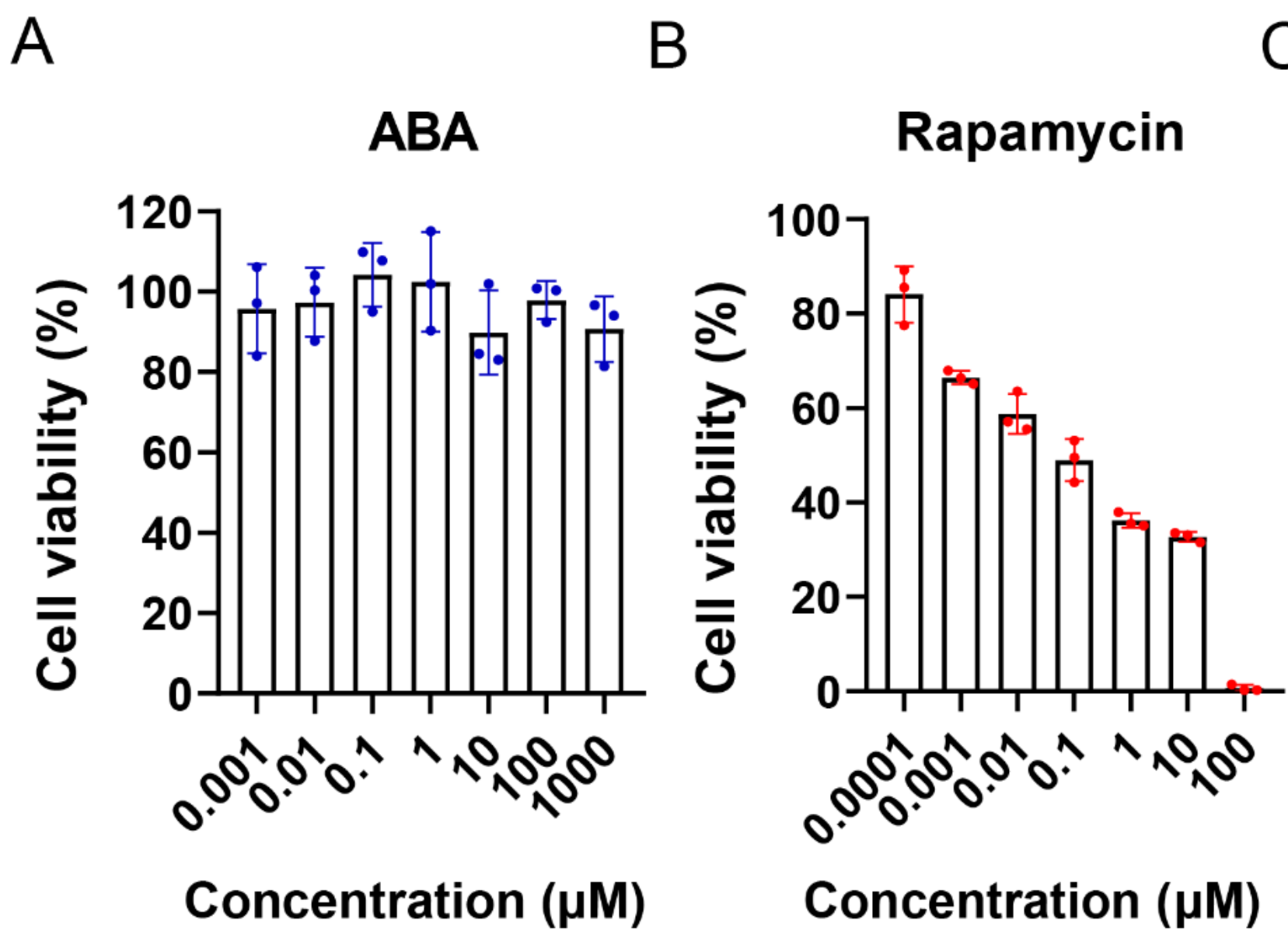

C

	IC_{50} (μM)
Rapamycin	0.716 ± 0.358
ABA	>1000

Figure S2. Cell viability analysis of ABA and rapamycin. (**A, B**) MTT assay of U2OS cells treated with increasing concentrations of ABA (**A**) or rapamycin (**B**). Cell viability is expressed as relative viability (% of untreated control). Data represent mean ± s.e.m. from three biological replicates (*n* = 4). (**C**) IC_{50} values of rapamycin and ABA in U2OS cells, determined by nonlinear regression analysis of dose-response curves using GraphPad Prism 9. Data represent mean ± s.e.m. from three independent biological replicates (*n* = 3).

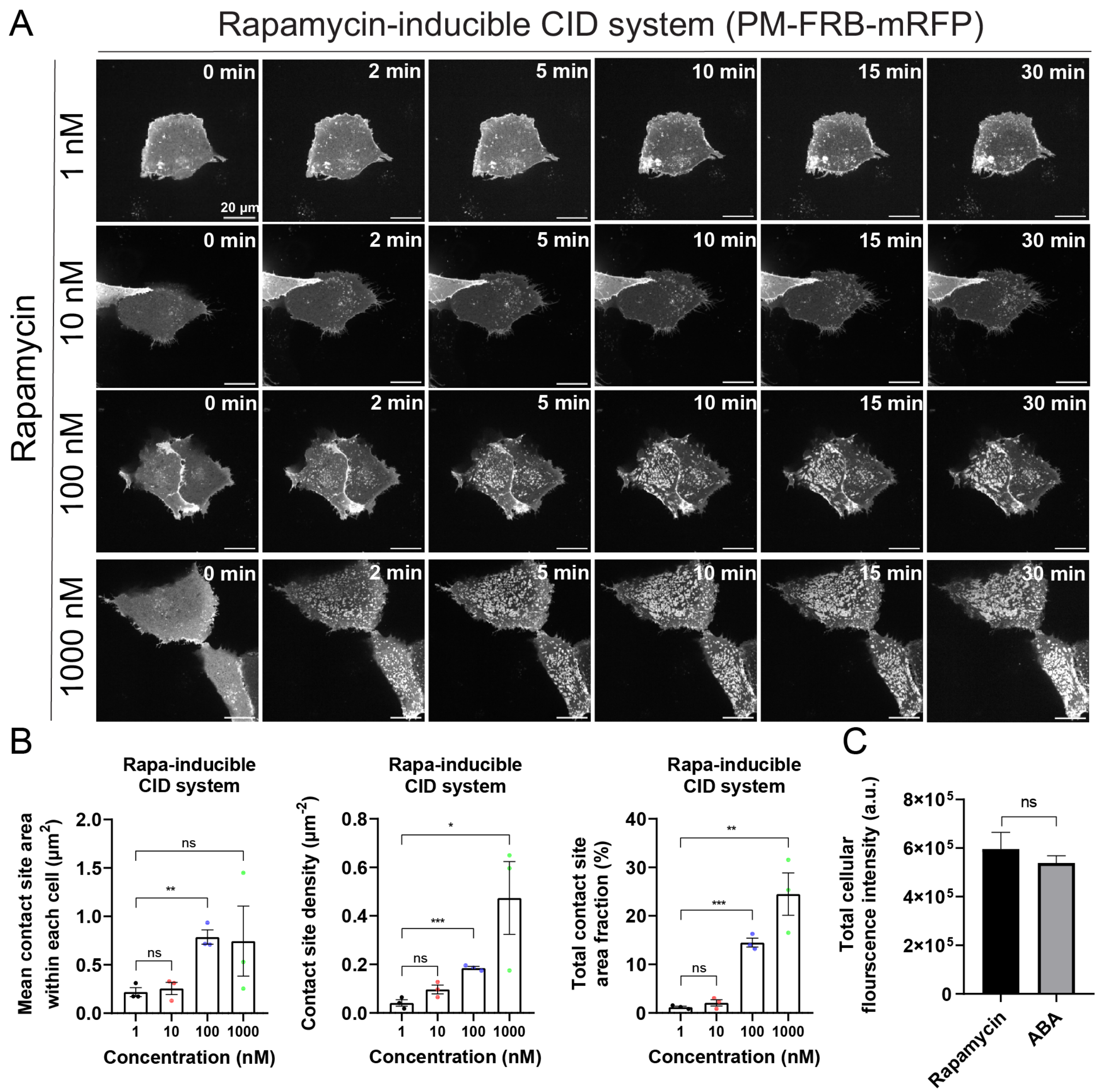

Figure S3. Quantitative analysis of the rapamycin-inducible CID system. (**A**) Representative time-course (0-30 min) and dose-response images of rapamycin-induced ER-PM contact site formation in U2OS cells. Cells were transfected with rapamycin-inducible CID construct and imaged by confocal microscopy 48 h after transfection. Scale bar: 20 µm. (**B**) Quantification of contact-site mean size, density, and area fraction at varying Rapamycin concentrations. Data represent mean ± s.e.m. (n = 3). (**C**) Total cellular fluorescence intensity was quantified in U2OS

cells expressing the ABA- and rapamycin-inducible CID systems. Data represent mean ± s.e.m. from three independent biological replicates ($n = 3$). P values were calculated using two-tailed unpaired Student's t tests: *$P < 0.05$, **$P < 0.01$, ***$P < 0.001$, ****$P < 0.0001$, and ns, not-significant.

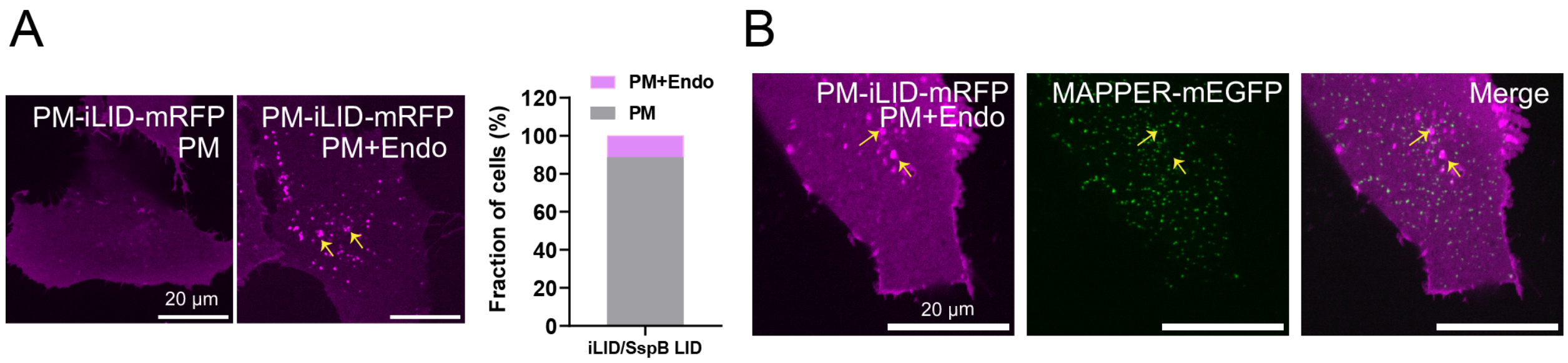

Figure S4. Subcellular localization of Lyn-iLID-mRFP in iLID/SspB LID system. (**A**) Left: representative confocal images showing two distinct localization patterns of Lyn-iLID-mRFP observed in iLID/SspB LID systems. "PM" indicates that Lyn-iLID-mRFP (i.e., PM-iLID-mRFP) is predominantly localized to the plasma membrane. "PM+Endo" indicates that Lyn-iLID-mRFP is localized to both the plasma membrane and endosomal vesicles. Yellow arrows indicate endosomal vesicles. Scale bar: 20 μm. Right: Quantification shows the fraction of U2OS cells exhibiting PM localization or PM+Endo localization in iLID/SspB LID system. (**B**) Representative confocal images of U2OS cells co-expressing the iLID/SspB system and MAPPER-mEGFP before blue light illumination. Scale bar: 20 μm. Yellow arrows indicate endosomal vesicles.

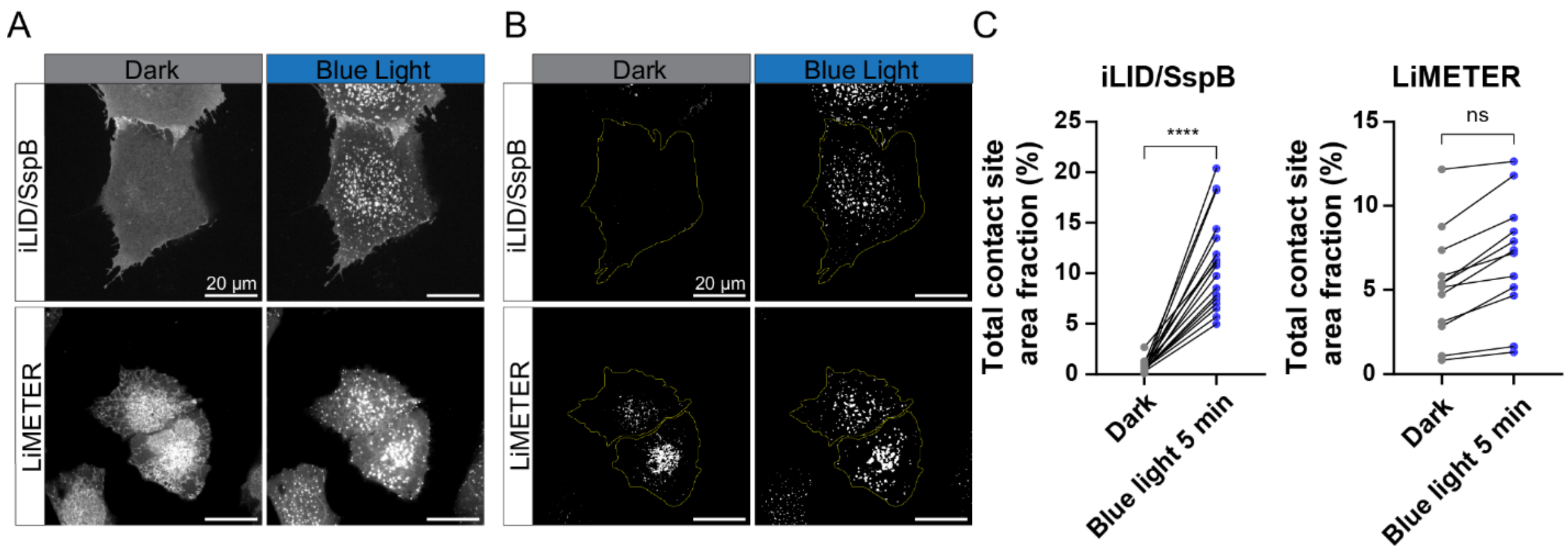

Figure S5. Comparison of the iLID/SspB LID and LiMETER systems. (**A**) Representative confocal images of the ventral surface of U2OS cells expressing iLID/SspB LID construct (mRFP channel) or LiMETER construct (GFP channel) before (left) and 5 min after (right) blue-light illumination. A 470-nm laser (~142 mW/cm^2, measured at the sample plane) was used to activate the iLID/SspB or LiMETER system, and GFP signals were simultaneously acquired for LiMETER. Scale bar: 20 µm. (**B**) Segmentation masks of the ventral surface of U2OS cells shown in Fig. S4A, generated by MATLAB-based image analysis to identify punctate ER-PM contact sites. These masks were used as the cell area for contact site quantification. (**C**) Quantification of total ER-PM contact site area fraction before and after blue-light illumination in U2OS cells expressing iLID/SspB (n = 17 cells) or LiMETER (n = 12 cells) construct, based on the ventral surface confocal images shown in Fig. S4A. P values were calculated using two-tailed unpaired Student's *t* tests: *$P < 0.05$, **$P < 0.01$, ***$P < 0.001$, ****$P < 0.0001$, and ns, not-significant.

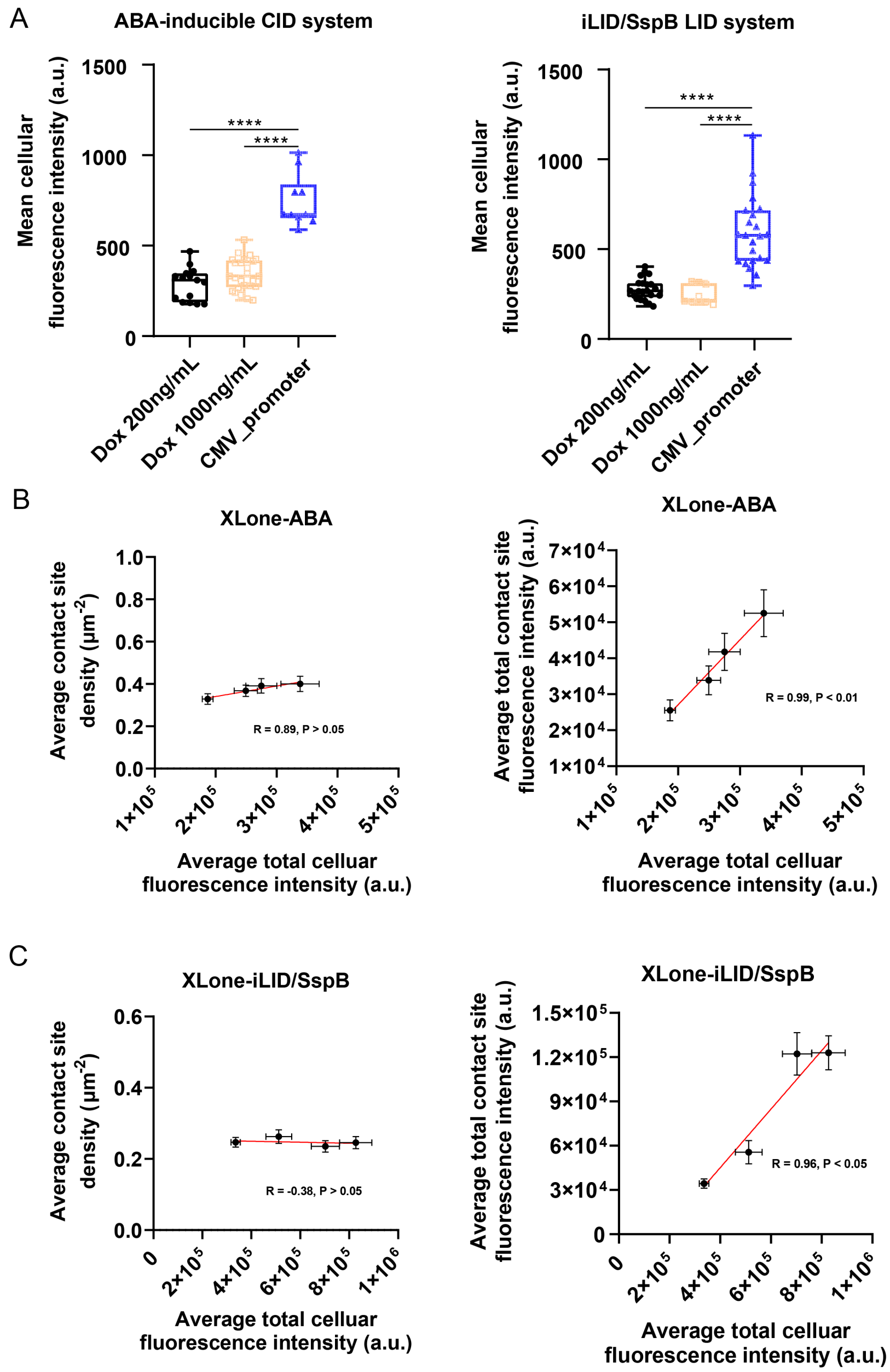

Figure S6. Expression-level analysis of the XLone-inducible ABA CID or iLID/SspB LID systems. (**A**) Mean cellular fluorescence intensity was quantified in cells expressing the ABA-inducible CID system (left) and the iLID/SspB LID system (right). For each inducible system, fluorescence intensity was analyzed in both CMV promoter-driven and XLone expression systems. P values were calculated using two-tailed unpaired Student's *t* tests: $*P < 0.05$, $**P < 0.01$, $***P < 0.001$, $****P < 0.0001$, and ns, not-significant. (**B**, **C**) Correlations between average total cellular

fluorescence intensity and ER-PM contact site parameters, including average contact site density (left) and average total contact site fluorescence intensity (right) in XLone-ABA-inducible CID (**B**) or XLone-iLID/SspB LID (**C**) systems. Pearson correlation coefficients (R) and corresponding *P* values were calculated using GraphPad Prism 9.

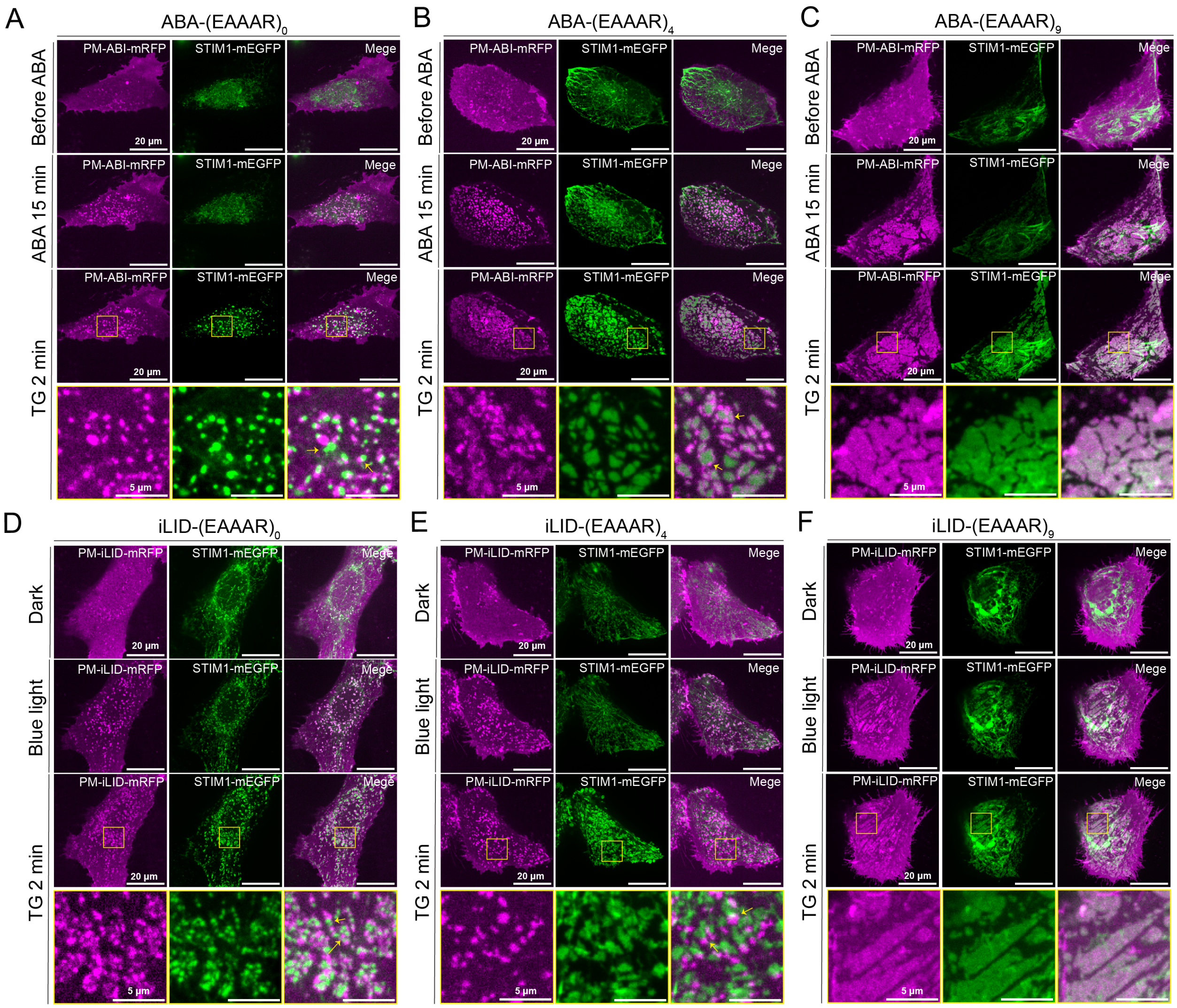

Figure S7. STIM1 recruitment in systems with varying linker lengths. Representative confocal images of U2OS cells from three independent experiments co-expressing STIM1-mEGFP and either an ABA-inducible CID system or an iLID/SspB LID system with varying spacer lengths. (**A**-**C**) Cells expressing ABA-inducible CID constructs with spacer lengths $(EAAAR)_0$ (**A**), $(EAAAR)_4$ (**B**), and $(EAAAR)_9$ (**C**) were first treated with 100 μM ABA for 15 min to induce ER-PM contact site formation, followed by treatment with 1 μM thapsigargin (TG) for 2 min to trigger STIM1 translocation. (**D**-**F**) Cells expressing iLID/SspB LID constructs with spacer lengths $(EAAAR)_0$ (**D**), $(EAAAR)_4$ (**E**), and $(EAAAR)_9$ (**F**) were first illuminated with blue light (71

mW/cm^2) for 5 min to induce contact site formation, followed by treatment with 1 μM TG for 2 min to induce STIM1 translocation. Yellow boxed regions in the TG-treated images are shown at higher magnification in the bottom panels. Yellow arrows in magnified views highlight the spatially exclusive localization between TG-induced STIM1 clusters and inducible contact sites. Scale bars: 20 μm (original view); 5 μm (magnified view).

References

(1) Zhang, S.; Chen, Y.; Xu, Z.; Yang, J.; Sun, R.; Wang, J.; Sun, Y.; Jiang, B.; Yang, X.; Tan, W. The PROTAC Selectively Degrading Bcl-xL Represents a Novel Hedgehog Pathway Inhibitor with Capacity of Combating Resistance to Smoothened Inhibitors While Sparing Bone Growth. *Theranostics* **2022**, *12* (17), 7476–7490. https://doi.org/10.7150/thno.75421.
